# Macrophage Dab1 Links Early Hypoxia to Adaptive Angiogenesis to Drive Peripheral Nerve Repair

**DOI:** 10.1002/advs.77705

**Published:** 2026-09-10

**Authors:** Xiongyao Zhou, Fan Zhang, Jiangbo Wang, Huiyuan Wang, Zongqiang Wang, Qianqian Wang, Chuikai Meng, Jialu Sun, Han Wang, Weiye Li, Ziyi Zhao, Le Qi, Xiaosong Gu, Shusen Cui, Rangjuan Cao

**Affiliations:** ^1^ Department of Hand and Foot Surgery China‐Japan Union Hospital of Jilin University Changchun Jilin China; ^2^ Key Laboratory of Peripheral Nerve Injury and Regeneration of Jilin Province Changchun Jilin China; ^3^ Department of Thyroid and Breast Surgery Yijishan Hospital The First Affiliated Hospital of Wannan Medical University Wuhu Anhui China; ^4^ Beihu Campus China‐Japan Union Hospital of Jilin University Changchun Jilin China; ^5^ Department of Wound Repair Plastic and Reconstructive Microsurgery China‐Japan Union Hospital of Jilin University Changchun Jilin China; ^6^ Key Laboratory of Neuroregeneration of Jiangsu and Ministry of Education Co‐innovation Center of Neuroregeneration Nantong University Nantong Jiangsu China

**Keywords:** angiogenesis, Dab1, hypoxia, macrophages, nerve regeneration

## Abstract

Effective nerve regeneration depends on the precise spatiotemporal coordination of immune responses and adaptive angiogenesis, often driven by injury‐induced hypoxia. However, how early hypoxic signals are sensed and translated into vascular niches remains poorly understood. Using peripheral nerve injury as a model, we show that hypoxia exerts stage‐specific functions dictating regenerative outcomes. Suppressing the early hypoxic response by premature oxygen supplementation lowers HIF‐1α, attenuates angiogenesis, and ultimately compromises long‐term regeneration, whereas prolonged hypoxia *ex vivo* causes axonal and myelin injury. Using spatiotemporal hypoxia mapping, genetic mouse models, and human nerve specimens, we identify Disabled‐1 (Dab1) as a previously unrecognized hypoxia‐responsive adaptor that is prominently induced in macrophages and links hypoxia to angiogenesis. Macrophage‐specific Dab1 deletion diminishes HIF‐1α/VEGF‐A signaling, impairs vascular remodeling, and delays nerve regeneration, whereas enhancing Dab1 signaling improves the regenerative microenvironment. Mechanistically, Dab1 sustains hypoxia‐associated HIF‐1α accumulation, partially through the Na^+^/H^+^ exchanger NHE1, maintaining a pro‐angiogenic program in macrophages. Furthermore, the clinically approved drug valproic acid promotes angiogenesis and functional recovery by enhancing macrophage Dab1 signaling within an early therapeutic window. Together, these findings identify macrophage Dab1 as a crucial regulator of vascular‐immune crosstalk, highlighting therapies that balance early hypoxic signaling with timely vascular reconstruction.

## Graphical abstract

Schematic diagram illustrating the Dab1‐linked hypoxia signaling pathway that accelerates angiogenesis and consequent nerve regeneration. Following PNI, a hypoxic microenvironment develops. Hypoxia induces macrophage Dab1 phosphorylation, resulting in NHE‐1 upregulation, HIF‐1α stabilization, and VEGF‐A expression. This cascade promotes angiogenesis in the injured nerve. VPA further amplifies this pathway, enhancing vascular remodeling to support functional recovery.

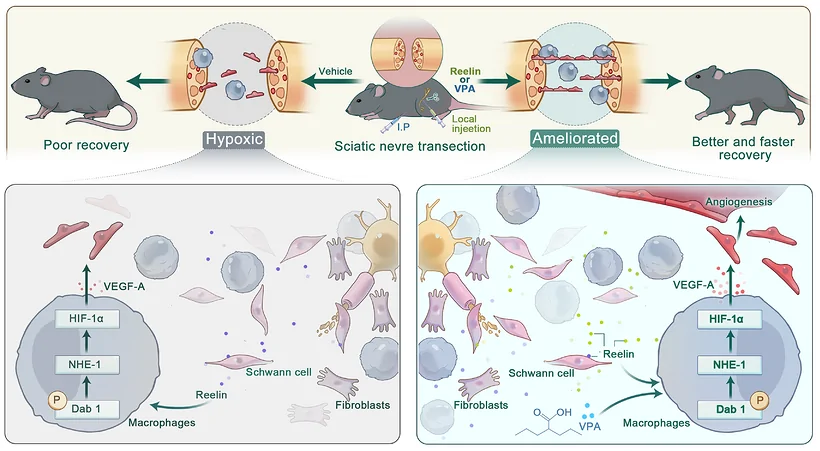

## 1 Introduction

Although peripheral nerves retain an intrinsic capacity for regeneration following injury, this process is often disrupted by various factors, resulting in unsatisfactory long‐term functional recovery in clinical practice [1, 2]. Accumulating evidence suggests that regenerative failure is not attributable to a defect at a single step, but rather arises from impaired reconstruction of the injury microenvironment [1, 3, 4]. Therefore, defining the mechanisms that govern the regenerative microenvironment is essential for developing effective therapeutic strategies after peripheral nerve injury (PNI).

The concept of coordinated neuro–vascular–immune repair has provided a more integrative framework for understanding the nerve regeneration microenvironment. Following PNI, hypoxia resulting from insufficient local perfusion can be sensed by multiple cell types, including endothelial cells, Schwann cells, and macrophages, thereby activating a hypoxia‐responsive transcriptional program centered on HIF‐1α. This program promotes the expression and secretion of pro‐angiogenic factors such as VEGF‐A, ultimately driving angiogenesis and vascular remodeling within the injured microenvironment [5, 6, 7]. Newly formed blood vessels not only provide metabolic and oxygen support, but also serve as structural “regenerative tracks” that guide the directed migration of repair Schwann cells and facilitate axonal elongation [5, 8, 9, 10]. Nevertheless, some studies have shown that improving oxygen supply enhances Schwann cell survival and vascular regeneration, leading to better repair outcomes, which suggests that oxygen insufficiency itself may restrict regeneration [11, 12]. Therefore, these findings raise a critical paradox: hypoxia may function either as an adaptive signal or as a detrimental stress during nerve repair, depending on the severity, duration, and cellular context of oxygen deprivation.

Among the cellular components of the injured nerve, macrophages are particularly well positioned to translate hypoxic stress into regenerative remodeling [13, 14]. In addition to their established roles in debris clearance and inflammatory resolution, macrophages actively regulate angiogenesis by producing pro‐vascular mediators and interacting with endothelial and glial compartments within the regenerating niche [15, 16]. Emerging evidence suggests that macrophages can sense local oxygen deprivation and initiate HIF‐1α‐dependent pro‐angiogenic responses [6, 17, 18]; however, the molecular mechanism that couples hypoxia sensing to pro‐regenerative vascular remodeling in these cells remains poorly defined in peripheral nerve injury. In particular, it remains unclear how early hypoxic signaling is maintained to support vascular remodeling without progressing into persistent maladaptive stress.

Disabled‐1 (Dab1), an adaptor protein best known for its role in developmental signaling, has also been reported to be involved in blood vessel formation in the central nervous system [19, 20]. Whether Dab1 contributes to regeneration‐associated vascular remodeling following PNI, and specifically, whether this process is mediated by macrophage‐driven hypoxic responses, remains largely unknown. In the present study, using a sciatic nerve transection model, we characterized the spatiotemporal changes in hypoxia after injury and investigated its role in vascular regeneration [5, 21]. Our data support the view that hypoxia exerts stage‐specific effects during peripheral nerve regeneration. We further identify Dab1 as a previously unrecognized regulator of the macrophage hypoxic response and provide evidence that Dab1‐dependent signaling contributes to HIF‐1α/VEGF‐A activation and vascular remodeling in the injured nerve. From a translational perspective, our findings demonstrate that the clinically approved mood stabilizer valproic acid (VPA) promotes angiogenesis and improves nerve regeneration during the critical early stage after injury by enhancing Dab1 signaling in macrophages. Together, these findings define a macrophage‐centered mechanism by which injury‐induced hypoxia is translated into regenerative vascular remodeling and support a stage‐specific repair strategy.

## 2 Results

### 2.1 Hypoxia as a Stage‐Specific Regulator of Axonal Regeneration Following Injury

**FIGURE 1:**
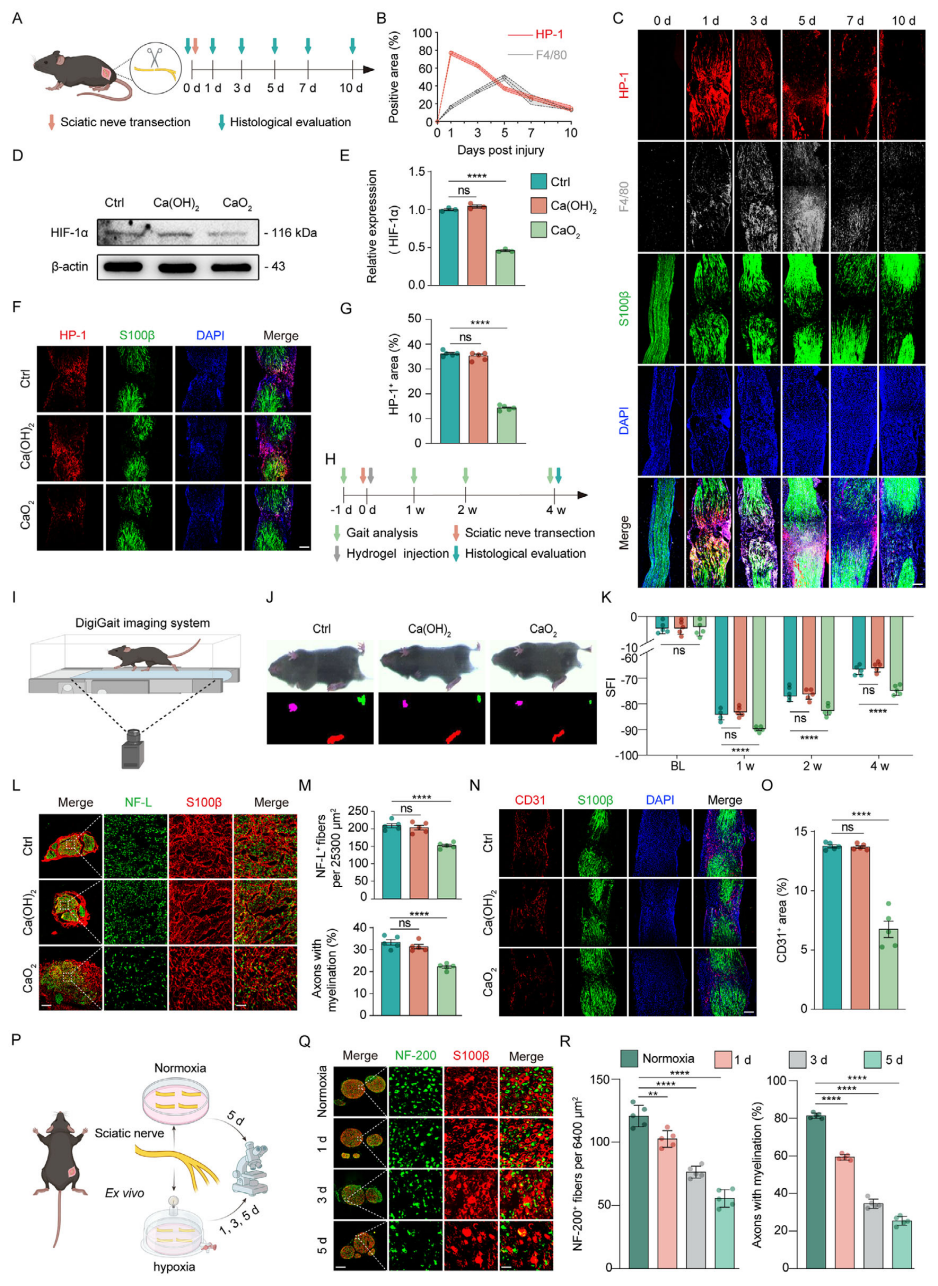
Hypoxia as a stage‐specific regulator of axonal regeneration following injury. (A) Schematic illustration of the experimental timeline following sciatic nerve transection. (B,C) Quantification and representative immunofluorescence images of longitudinal sciatic nerve sections at 0, 1, 3, 5, 7, and 10 days post‐surgery, stained with Hypoxyprobe‐1 (HP‐1), F4/80, S100β, and DAPI. Scale bar = 200 µm, *n* = 5 mice. (D) Representative Western blot images of HIF‐1α protein levels in nerves treated with Ctrl, Ca(OH)_2_, or CaO_2_‐loaded hydrogel, with β‐actin as a loading control. (E) Quantification of relative expression of HIF‐1α in D (*n* = 3). (F) Representative immunofluorescence images of longitudinal sciatic nerve sections in the indicated treatment groups 5 days post‐surgery. Scale bar = 200 µm. (G) Quantification of HP‐1^+^ hypoxic area in F (*n* = 5 mice). (H) Schematic illustration of the experimental timeline of hydrogel injection and analysis. (I) The DigiGait imaging system used to evaluate gait performance post‐surgery. (J) Representative footprint images of mice in Ctrl, Ca(OH)_2_, and CaO_2_ groups at 4 weeks post‐surgery. (K) Quantification of the SFI at 1, 2, and 4 weeks post‐surgery (*n* = 5 mice). (L) Representative immunofluorescence images of regenerating nerves approximately 5 mm distal to the injury site under Ctrl, Ca(OH)_2_, or CaO_2_ treatment conditions, stained for NF‐L and S100β. Scale bars = 200 or 20 µm. (M) Quantification of NF‐L^+^ fiber density and the percentage of myelinated axons in L (*n* = 5 mice). (N) Representative immunofluorescence images of longitudinal sciatic nerve sections at 5 days post‐surgery, stained for CD31, S100β, and DAPI. Scale bars = 200 µm. (O) Quantification of CD31^+^ vascular area in nerves in N (*n* = 5 mice). (P) Schematic illustration of the ex vivo sciatic nerve culture under normoxic or hypoxic conditions. (Q) Representative images of axonal degeneration under normoxic conditions at 5 days and under hypoxic conditions at 1, 3, and 5 days ex vivo. (R) Quantification of NF‐200^+^ fiber density and the percentage of myelinated axons in Q (*n* = 5). Data are presented as mean ± SEM. Statistical analysis: two‐way ANOVA (K) or one‐way ANOVA (E, G, M, O, and R) with Bonferroni's post hoc test. Statistical significance was determined as indicated (^**^ *p* < 0.01, ^****^ *p* < 0.0001; ns, not significant).

PNI causes immediate local hypoxia due to vascular damage, but its temporal dynamics remain poorly defined. We therefore used the Hypoxyprobe‐1 (HP‐1) assay to assess hypoxia in nerve tissues at multiple time points after nerve injury [22, 23, 24], with F4/80^+^ macrophage infiltration as a reference (Figure 1A). Macrophage accumulation increased progressively, peaking at day 5 and remaining elevated through day 7, consistent with a prior report [25]. In contrast, hypoxia peaked as early as day 1 post‐injury and remained elevated through day 7 (Figure 1B,C).

Previous studies have reported that, following PNI, hypoxia activates HIF‐1α–dependent transcriptional programs, thereby upregulating pro‐angiogenic mediators and promoting post‐injury revascularization [8, 15]. However, whether hypoxia is an obligate prerequisite for peripheral nerve repair remains unclear. Following sciatic nerve transection, we locally applied a Pluronic F‐127 hydrogel loaded with calcium peroxide (CaO_2_) nanoparticles to increase tissue oxygen content [26, 27, 28]. At day 5 post‐surgery, hypoxia was assessed using the Hypoxyprobe‐1. Compared with the calcium hydroxide (Ca(OH)_2_) treatment, CaO_2_ nanoparticles markedly reduced the Hypoxyprobe‐1^+^ hypoxic area at the lesion site and downregulated HIF‐1α protein levels (Figure 1D–G). Gait analysis at 1, 2, and 4 weeks postoperatively revealed that, at 4 weeks, the CaO_2_‐treated group displayed reduced paw–ground contact area on heatmaps (Figure 1H–J) and a significantly decreased sciatic functional index (SFI) relative to the control (Ctrl) and Ca(OH)_2_ groups (Figure 1K). Transverse sections collected 5 mm distal to the injury site and stained for NF‐L (axons) and S100β (myelin) showed fewer regenerating axons and reduced myelination after CaO_2_ treatment (Figure 1L,M). To determine whether this impaired regeneration was associated with altered early angiogenesis, we stained day‐5 nerves for CD31 and observed a lower proportion of CD31^+^ area in the CaO_2_‐treated group (Figure 1N,O). Collectively, these findings suggest that hypoxia is required for angiogenesis after PNI and, consequently, for subsequent axonal regeneration.

Apart from blood vessels, neural components such as axons and myelinating Schwann cells are also exposed to hypoxia following PNI [29, 30]. However, whether hypoxia directly impacts the injured sciatic nerve tissue, and whether this is beneficial or detrimental, remains unclear. To address this, we isolated sciatic nerve explants and maintained them ex vivo [31]. Explants were cultured under normoxia (21% O_2_) for 5 days or under hypoxia (1% O_2_) for 1, 3, or 5 days, followed by transverse sectioning (Figure 1P). Axons were labeled with NF‐200 and myelin with S100β. Compared with the normoxic culture, hypoxia caused a significant reduction in axonal density and myelination as early as day 1, with progressively more severe pathological changes observed after prolonged exposure (Figure 1Q,R). Collectively, these data indicate that hypoxia acts as a double‐edged modulator of the regenerative microenvironment.

### 2.2 Dab1 Is a Hypoxia‐Responsive Protein Following PNI

**FIGURE 2:**
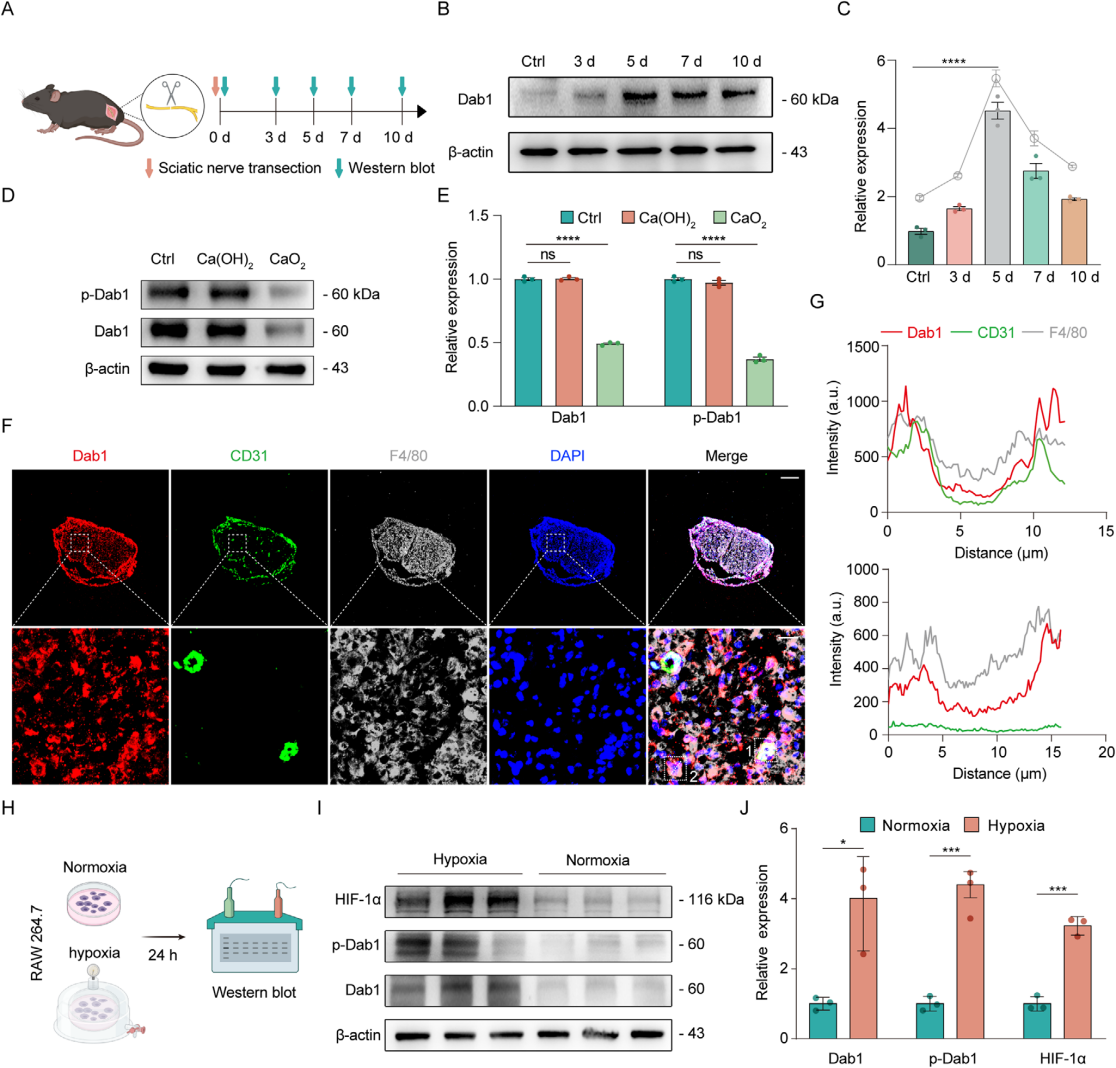
Dab1 is a hypoxia‐responsive protein following PNI. (A) Schematic illustration of the experimental design. (B) Representative Western blot images of Dab1 protein levels in sciatic nerves at 0 (ctrl), 3, 5, 7, and 10 days post‐surgery with β‐actin as a loading control. (C) Quantitative analysis of relative Dab1 protein levels in B (*n* = 3). (D) Representative Western blot images of p‐Dab1 and Dab1 in transected sciatic nerves treated with Ctrl, Ca(OH)_2_, or CaO_2_. (E) Quantification of relative expression of Dab1 and p‐Dab1 in D (*n* = 3). (F) Representative immunofluorescence images of sciatic nerve cross‐sections stained for Dab1, CD31, F4/80, and DAPI. Scale bars = 200 or 20 µm. (G) Line‐scan intensity profiles showing the spatial overlap of Dab1 with CD31 and F4/80 along the indicated regions in F (upper, region 1; lower, region 2). (H) Schematic illustration of RAW 264.7 macrophages cultured under normoxic or hypoxic conditions for analysis. (I) Representative Western blot images of HIF‐1α, p‐Dab1, and Dab1 in RAW 264.7 macrophages cultured under hypoxic or normoxic conditions. (J) Quantification of relative expression of HIF‐1α, p‐Dab1, and Dab1 normalized to β‐actin under normoxic and hypoxic conditions in I (*n* = 3). Data are presented as mean ± SEM. Statistical analysis: one‐way ANOVA with Tukey's post hoc test (C and E) and unpaired two‐tailed Student's *t*‐test (J), as indicated. Statistical significance was determined as indicated (^***^ *p* < 0.001, ^****^ *p* < 0.0001; ns, not significant).

Given that hypoxia‐driven HIF‐1α activation promotes angiogenesis and supports axonal regeneration, whereas prolonged hypoxia can induce neural degeneration and impair repair, we asked whether direct activation or selective potentiation of HIF‐1α signaling could enhance revascularization and thereby shorten the hypoxic period. GEO‐based transcriptomic analyses identified Dab1 as an upregulated gene following nerve injury (GSE45376; Figure S1A) [32], and Dab1 has been reported to regulate endothelial cell migration and promote angiogenesis in the central nervous system [19]; however, its role in peripheral nerve regeneration remains undefined. We therefore examined Dab1 expression after sciatic nerve transection (Figure 2A). Western blot analysis showed that Dab1 was strongly induced, peaking at day 5 (∼4.5‐fold; Figure 2B,C). Intriguingly, Dab1 and phosphorylated Dab1 (p‐Dab1) were markedly reduced in CaO_2_‐treated sciatic nerve tissues compared with the Ca(OH)_2_‐treated and untreated controls (Figure 2D,E), indicating that Dab1 expression is hypoxia‐dependent.

Next, we assessed the cellular localization of Dab1 in peripheral nerve tissue. At day 5 after sciatic nerve transection, transverse sciatic nerve sections were subjected to co‐immunofluorescence staining for F4/80, CD31, S100β, and NF‐200. Dab1 was mainly detected in macrophages and endothelial cells (Figure 2F,G; Figure S1B–E). Given that (1) a large number of macrophages are recruited after PNI [33]; (2) macrophage infiltration kinetics closely mirrored the temporal pattern of Dab1 upregulation (Figures 1B,C and 2B,C); and (3) macrophages play an important role under hypoxic conditions [13], we hypothesized that macrophage Dab1 is a hypoxia‐responsive protein. To test this, RAW 264.7 cells were cultured under hypoxic or normoxic conditions. After 24 h, Western blot analysis confirmed HIF‐1α induction under hypoxia and showed significant increases in Dab1 and p‐Dab1 levels (Figure 2H–J). Collectively, these results indicate that hypoxia directly promotes Dab1 expression and activation in macrophages.

### 2.3 Macrophage Dab1 Mediates Hypoxia‐Induced HIF‐1α/VEGF‐A Expression and Angiogenesis

**FIGURE 3:**
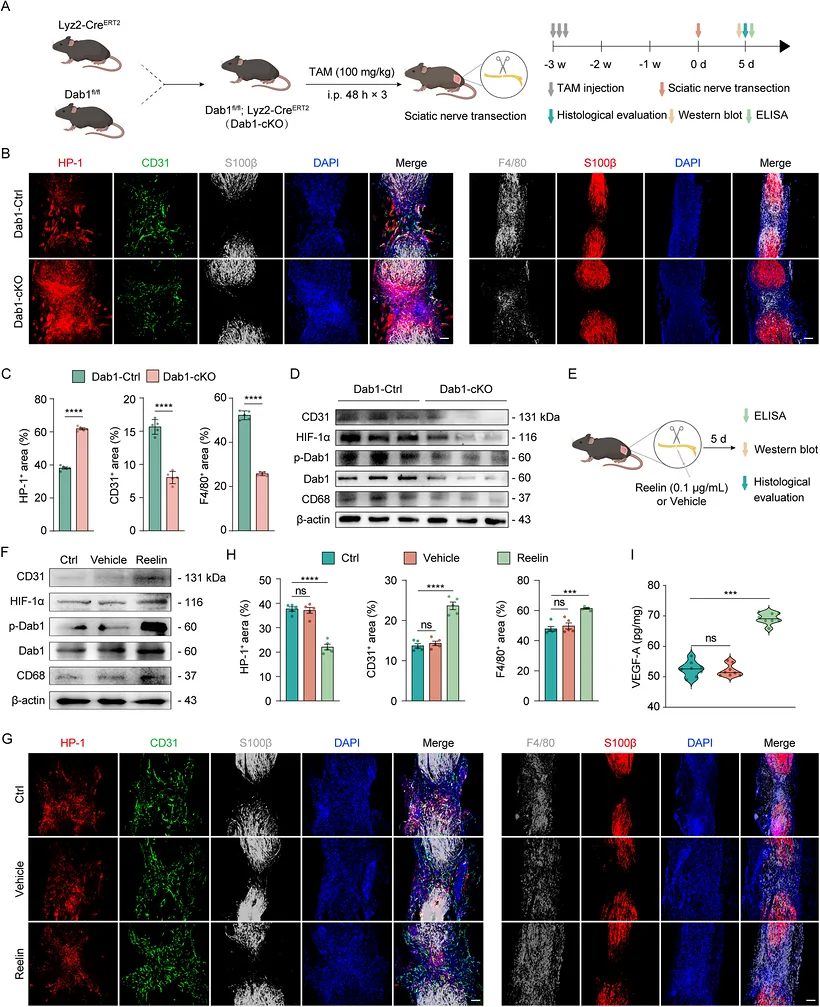
Macrophage Dab1 mediates hypoxia‐induced HIF‐1α/VEGF‐A expression and angiogenesis. (A) Schematic of the generation of macrophage‐specific Dab1 conditional knockout mice (Dab1^fl/fl^; Lyz2‐Cre^ERT2^) and the experimental timeline. (B) Representative immunofluorescence images of longitudinal sciatic nerve sections from Dab1‐Ctrl and Dab1‐cKO mice. Scale bar = 200 µm. (C) Quantification of HP‐1^+^ hypoxic area, CD31^+^ vascular area, and F4/80^+^ area in B (*n* = 5 mice). (D) Representative Western blot images showing protein levels of CD31, HIF‐1α, p‐Dab1, Dab1, and CD68 in sciatic nerves from Dab1‐Ctrl and Dab1‐cKO mice, with β‐actin as a loading control. Statistical analyses corresponding to this figure are provided in Figure S2C. (E) Schematic illustration of sciatic nerve transection followed by local administration of Reelin or vehicle. (F) Representative Western blot images of CD31, HIF‐1α, p‐Dab1, Dab1, and CD68 in sciatic nerves from Ctrl, vehicle‐treated, or 0.1 µg/mL Reelin‐treated groups. Statistical analyses corresponding to this figure are provided in Figure S2E. (G) Representative immunofluorescence images of longitudinal sciatic nerve sections stained with HP‐1, CD31, S100β, and DAPI, or F4/80, S100β, and DAPI. Scale bar = 200 µm. (H) Quantification of HP‐1^+^ hypoxic area, CD31^+^ vascular area, and F4/80^+^ area in G (*n *= 5 mice). (I) VEGF‐A secretion in sciatic nerves from Ctrl, vehicle‐treated, and Reelin‐treated groups (*n* = 8 from 4 mice). Data are presented as mean ± SEM. Statistical analysis: one‐way ANOVA with Tukey's post hoc test (H,I) and unpaired two‐tailed Student's *t‐*test (C), as indicated. Statistical significance was determined as indicated (^***^ *p* < 0.001, ^****^ *p* < 0.0001; ns, not significant).

To determine whether hypoxia‐induced Dab1 upregulation and activation in macrophages are required for angiogenesis, we generated macrophage‐specific conditional Dab1 knockout mice (Dab1^fl/fl^; Lyz2‐Cre^ERT2^, Dab1‐cKO), using Dab1^fl/fl^ littermates as controls (Dab1‐Ctrl). Three weeks before sciatic nerve transection, tamoxifen was administered every other day (100 mg/kg) for three injections (Figure 3A). At day 5 post‐injury, immunofluorescence staining for Dab1 and F4/80 showed the loss of Dab1 signal in F4/80^+^ macrophages in Dab1‐cKO mice (Figure S2A,B), confirming efficient deletion. On longitudinal sections, CD31 and Hypoxyprobe‐1 staining revealed markedly reduced CD31^+^ neovascularization and a significantly expanded hypoxic area in Dab1‐cKO nerves. Consistently, F4/80 staining showed decreased macrophage infiltration (Figure 3B,C). Western blot analysis also demonstrated reduced Dab1 and p‐Dab1 (from non‐macrophage cells), along with decreased CD31 and CD68 expression. Notably, HIF‐1α levels were also significantly reduced in Dab1‐cKO nerves (Figure 3D; Figure S2C). Given that macrophage HIF‐1α promotes VEGF‐A secretion [34, 35], we next assessed VEGF‐A expression by ELISA. The results showed a marked reduction of VEGF‐A in Dab1‐cKO sciatic nerves 5 days after surgery (Figure S2D). Together, these results indicate that hypoxia‐induced Dab1 in macrophages is required to drive HIF‐1α/VEGF‐A expression and angiogenesis, and that Dab1 loss impairs macrophage recruitment.

We next sought to determine whether activation of Dab1 could enhance HIF‐1α expression, accelerate angiogenesis at this critical stage, and thereby accelerate the resolution of tissue hypoxia. Reelin, a canonical upstream ligand of Dab1, is a secreted glycoprotein that recruits and activates cytoplasmic Dab1 [36]. We established a sciatic nerve transection model in WT mice and administered local treatment with Reelin or vehicle (Figure 3E). On day 5 postoperatively, Western blot analysis of injured sciatic nerve tissue revealed that Reelin enhanced the levels of both total and p‐Dab1, which were accompanied by elevated CD31, HIF‐1α, and CD68 expression (Figure 3F; Figure S2E). Immunofluorescence staining of longitudinal sciatic nerve sections demonstrated that Reelin significantly increased CD31^+^ endothelial area while markedly reducing Hypoxyprobe‐1^+^ hypoxic area at the injury site (Figure 3G,H). Reelin also increased the recruitment of F4/80^+^ macrophages and enhanced VEGF‐A secretion (Figure 3G–I). To rule out injection‐induced nerve injury, we performed ATF3 staining on dorsal root ganglia (DRG) at 1 day post‐surgery to quantify stressed neurons [37]. We found that the injection procedure itself caused no significant neuronal stress (Figure S2F,G). Together, these results suggest that Dab1 activation promotes the HIF‐1α/VEGF‐A signaling axis, improves the local hypoxic microenvironment, and enhances both angiogenesis and macrophage recruitment.

### 2.4 Activation of Macrophage Dab1 Orchestrates a Hypoxia‐Like Angiogenic Response via the HIF‐1α/VEGF‐A Axis

**FIGURE 4:**
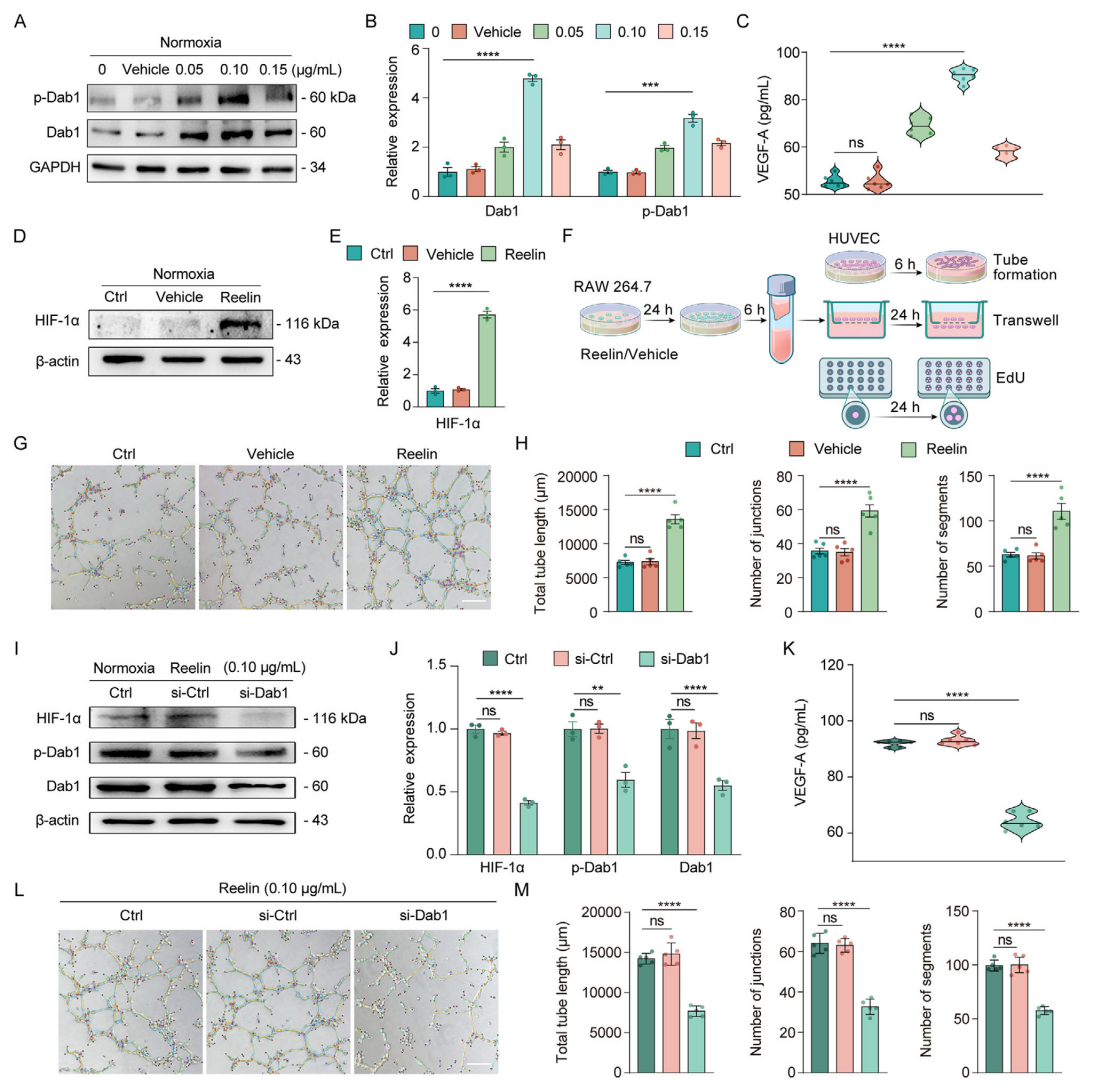
Activation of macrophage Dab1 orchestrates a hypoxia‐like angiogenic response via the HIF‐1α/VEGF‐A axis. (A) Representative Western blot images of p‐Dab1 and Dab1 in RAW 264.7 macrophages treated with vehicle or increasing concentrations of Reelin (0.05, 0.10, 0.15 µg/mL). (B) Quantification of relative expression of p‐Dab1 and Dab1 in A (*n* = 3). (C) ELISA analysis of VEGF‐A secretion (*n* = 6). (D) Representative Western blot images of HIF‐1α in RAW 264.7 macrophages cultured under normoxia and treated with Ctrl, vehicle, or Reelin, with β‐actin as a loading control. (E) Quantification of relative expression of HIF‐1α in D (*n* = 3). (F) Schematic illustration of the experimental workflow. (G) Representative images of tube formation by HUVECs cultured with conditioned media from the indicated groups under normoxia. Scale bar = 20 µm. (H) Quantification of total tube length, number of junctions, and number of segments from tube formation assays in G (*n* = 5). (I) Representative Western blots of HIF‐1α, p‐Dab1, and Dab1 in Reelin‐treated RAW 264.7 macrophages transfected with si‐Ctrl or si‐Dab1. (J) Quantification of relative expression of HIF‐1α, p‐Dab1, and Dab1 in I (*n *= 3). (K) VEGF‐A secretion measured by ELISA in the indicated groups (*n* = 6). (L) Representative images of tube formation by HUVECs cultured with conditioned media from Reelin‐treated RAW 264.7 macrophages transfected with the indicated siRNAs. Scale bar = 20 µm. (M) Quantification of total tube length, number of junctions, and number of segments from tube formation assays in L (*n* = 5). Data are presented as mean ± SEM. Statistical analysis: one‐way ANOVA with Tukey's post hoc test as indicated. Statistical significance was determined as indicated (^**^ *p* < 0.01, ^***^ *p* < 0.001, ^****^ *p* < 0.0001; ns, not significant).

We next asked whether, under normoxic conditions, activating Dab1 in macrophages is sufficient to promote angiogenesis via the HIF‐1α/VEGF‐A pathway. To determine the optimal stimulation conditions, RAW 264.7 macrophages were exposed to varying concentrations of Reelin under normoxia. We found that 0.1 µg/mL Reelin maximally induced the transcription of *Dab1* and *VEGF‐A* (Figure S3A), triggered the strongest Dab1 phosphorylation and VEGF‐A secretion (Figure 4A–C). Consistently, 0.1 µg/mL Reelin increased HIF‐1α expression under normoxia (Figure 4D,E). Further time‐course evaluation demonstrated a rapid upregulation of both p‐Dab1 and HIF‐1α within 15 min, maintaining elevated levels up to 24 h. Consequently, treatment with 0.1 µg/mL Reelin for 24 h was selected for subsequent experiments. Intriguingly, total Dab1 protein remained unchanged at 15 min but increased at 60 min, with elevated levels sustained through 24 h. RT‐qPCR results recapitulated this expression pattern, which suggests a transcriptional promotion of *Dab1* by Reelin (Figure S3B–D).

To assess the pro‐angiogenic activity of macrophage‐derived factors, RAW 264.7 cells were stimulated with 0.1 µg/mL Reelin for 24 h, followed by medium replacement and an additional 6 h of culture. Supernatants were concentrated approximately 10‐fold [38] and applied to HUVECs to evaluate tube formation, migration, and proliferation (Figure 4F). In a Matrigel tube formation assay [39], concentrated conditioned media from Reelin‐treated macrophages significantly increased total tube length, junction number, and segment number compared with vehicle and blank controls (Figure 4G,H). Additionally, Reelin‐conditioned media increased HUVEC migration and proliferation (Figure S3E–H).

We next tested whether Dab1 is required for this normoxic HIF‐1α/VEGF‐A induction and pro‐angiogenic effect. RAW 264.7 cells were first stimulated with 0.1 µg/mL Reelin for 24 h, and subsequently transfected with si‐Dab1 or si‐Ctrl for an additional 24 h. Western blot analysis revealed that si‐Dab1 reduced Dab1 and p‐Dab1 and concomitantly decreased HIF‐1α expression (Figure 4I,J). ELISA revealed reduced VEGF‐A in conditioned media from si‐Dab1‐treated cells (Figure 4K). When these conditioned media were concentrated and applied to HUVECs, tube formation was inhibited, with reduced total tube length, the number of junctions, and segments (Figure 4L–M); HUVEC migration and proliferation were likewise decreased (Figure S3I–L). These results indicate that Dab1 knockdown suppresses HIF‐1α/VEGF‐A expression and impairs endothelial angiogenic behaviors, even under normoxic conditions.

After PNI, recruited macrophages at the lesion site secrete VEGF‐A, driving angiogenesis that supports Schwann cell–guided axonal regeneration [5, 33, 40, 41]. Functionally, Reelin activation enhanced RAW 264.7 migration and proliferation (Figure S3M–P). In contrast, Dab1 knockdown after Reelin stimulation markedly reduced macrophage migration and proliferation (Figure S3Q–T). Immunofluorescence showed that Dab1 expression was markedly increased after 24 h of Reelin stimulation, co‐localized with F4/80, and was associated with a polarized macrophage morphology (Figure S3U,V). Together, these data support a role for macrophage Dab1 in promoting angiogenesis and peripheral nerve repair via HIF‐1α/VEGF‐A signaling, as evidenced by consistent in vitro and in vivo findings.

### 2.5 NHE1 Functions as a Downstream Effector of Dab1 in Macrophage HIF‐1α Signaling

**FIGURE 5:**
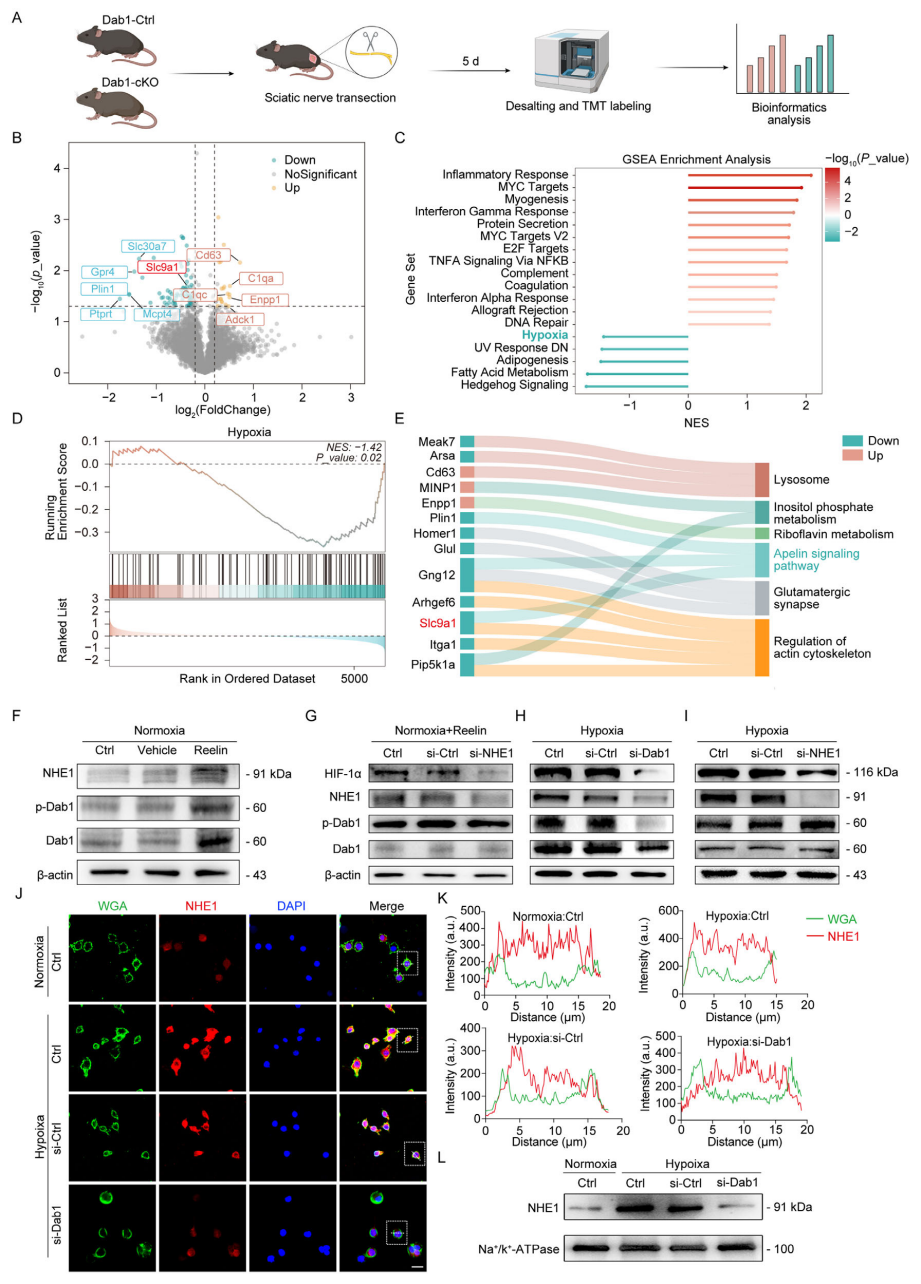
NHE1 functions as a downstream effector of Dab1 in macrophage HIF‐1α signaling. (A) Schematic diagram of the proteomic analysis. (B) Volcano plot summarizing differential protein abundance, with significantly increased and decreased proteins highlighted. (C) GSEA of pathways differentially represented between groups, shown as NES with significance encoded by –log_10_ (*p*‐value). (D) Representative GSEA enrichment plot for the hypoxia hallmark gene set (NES = 1.42; *p* = 0.02). (E) Sankey diagram linking representative differentially expressed proteins to enriched functional pathways. (F) Representative Western blot images showing NHE1, p‐Dab1, and Dab1 protein levels in RAW 264.7 macrophages cultured under the indicated conditions. Statistical analyses corresponding to this figure are provided in Figure S4B. (G–I) Representative Western blots of HIF‐1α, NHE1, p‐Dab1, and Dab1 under normoxia with Reelin treatment after si‐Ctrl or si‐NHE1 transfection, and under hypoxia after si‐Dab1 or si‐NHE1 transfection. Statistical analyses corresponding to this figure are provided in Figure S4C–E. (J) Representative immunofluorescence images showing WGA (labeling the plasma membrane), NHE1, and DAPI in the indicated groups. Scale bar = 20 µm. (K) Line‐scan intensity profiles showing the spatial overlap of WGA and NHE1 signals along the indicated regions in J. (L) Representative Western blot images showing plasma membrane NHE1 protein levels in RAW 264.7 macrophages cultured under normoxia or hypoxia with si‐NHE1 or si‐Ctrl. Na^+^/K^+^‐ATPase as a loading control. Statistical analyses corresponding to this figure are provided in Figure S4F.

To further define how macrophage Dab1 regulates the hypoxia phenotype, we established a sciatic nerve transection model in Dab1‐cKO and Dab1‐Ctrl mice, and performed TMT‐based quantitative proteomics on day 5 post‐surgery (Figure 5A). We identified 93 differentially expressed proteins, with 20 upregulated and 73 downregulated (Figure 5B). GSEA of the proteomic data revealed significant suppression of hypoxia‐related biological processes in Dab1‐cKO nerves (Figure 5C,D), further supporting a tight link between Dab1 and hypoxic responses. KEGG analysis showed suppression of the apelin signaling pathway (Figure 5E), which is essential for angiogenesis and vascular homeostasis [42, 43], suggesting that Dab1 deficiency disrupts pro‐revascularization networks. GO enrichment analysis also revealed perturbations in multiple angiogenesis‐related pathways (e.g., endothelial cell migration and blood vessel formation), along with inflammatory pathways including NIK/NF‐κB (Figure S4A). Together, these data suggest that Dab1 deficiency reshapes the regenerative microenvironment by modulating hypoxic responses, vascular regeneration, and inflammation‐associated signaling.

Notably, SLC9A1 (also known as the Na^+^/H^+^ exchanger 1, NHE1), a key regulator of intracellular pH homeostasis [44], was among the significantly downregulated proteins (Figure 5B). NHE1 has been shown to promote HIF‐1α stability and activity through the inhibition of prolyl hydroxylase (PHD) and activation of PI3K/Akt/mTOR signaling [45, 46]. We therefore hypothesized that NHE1 mediates Dab1‐dependent regulation of HIF‐1α. To test this, RAW 264.7 macrophages were stimulated with Reelin for 24 h under normoxia. Western blot confirmed that Reelin increased HIF‐1α expression concomitant with the upregulation of Dab1, p‐Dab1, and NHE1 (Figure 5F; Figure S4B). Following Reelin activation under normoxia, siRNA‐mediated knockdown of NHE1 (si‐NHE1) did not significantly alter Dab1 or p‐Dab1 levels but blunted the induction of HIF‐1α (Figure 5G; Figure S4C), indicating that NHE1 acts downstream of Dab1 to sustain HIF‐1α. We next validated this mechanism under hypoxia. Dab1 knockdown (si‐Dab1) reduced p‐Dab1 and NHE1, and markedly decreased HIF‐1α (Figure 5H; Figure S4D). Conversely, si‐NHE1 under hypoxia did not affect Dab1 or p‐Dab1, but reduced HIF‐1α (Figure 5I; Figure S4E), supporting NHE1‐dependent maintenance of HIF‐1α. Furthermore, immunofluorescence and Western blot analysis of membrane protein fractions revealed that Dab1 knockdown markedly reduced the plasma membrane localization of NHE1 under hypoxic conditions (Figure 5I,J; Figure S4F). Collectively, these results indicate that NHE1 functions downstream of Dab1 as a key determinant of HIF‐1α maintenance, with minimal feedback on Dab1 itself.

### 2.6 Dab1 Promotes Vascular Regeneration via the NHE1/HIF‐1α Pathway

**FIGURE 6:**
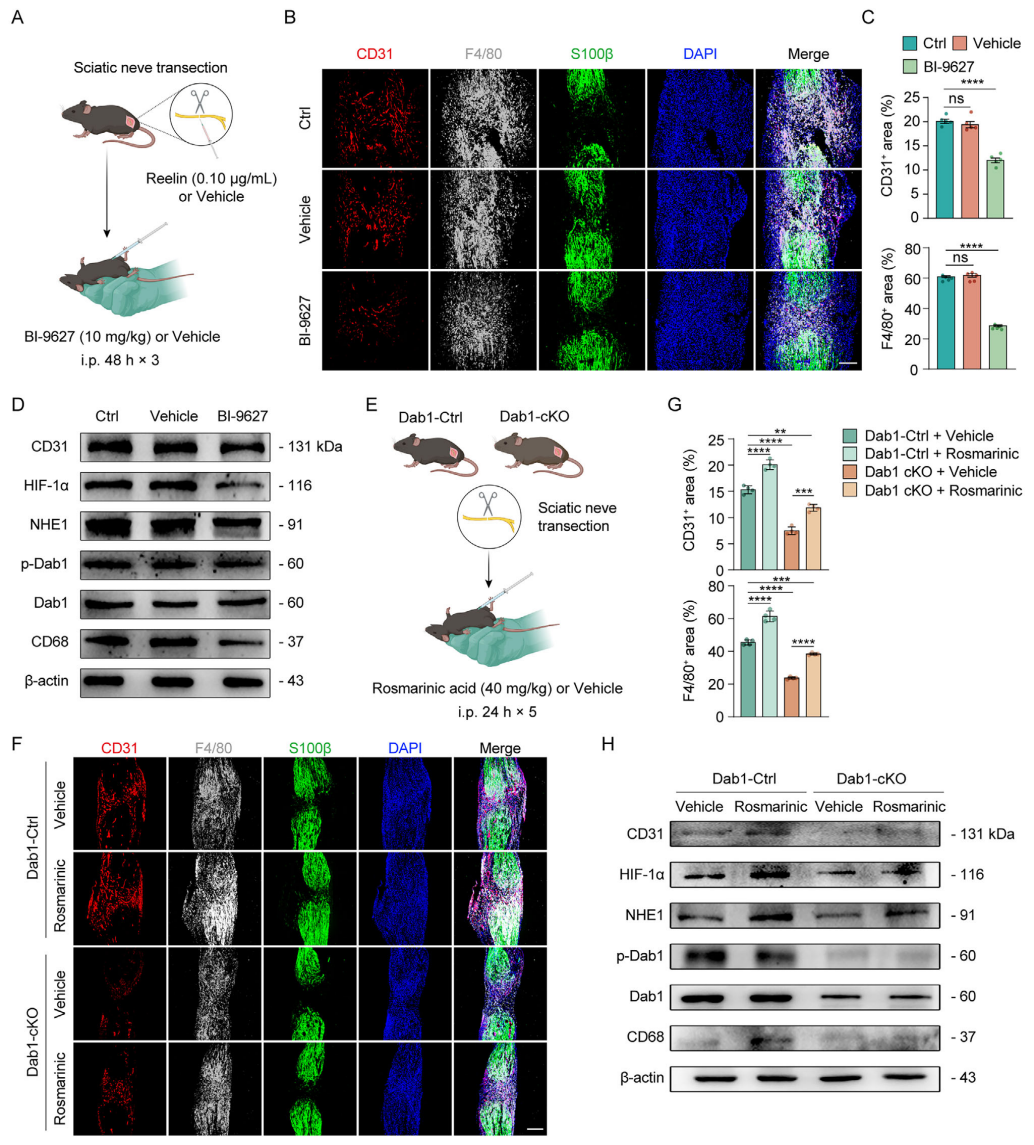
Dab1 promotes vascular regeneration via the NHE1/HIF‐1α pathway. (A) Schematic of the sciatic nerve transection model with local Reelin delivery and intraperitoneal injection of the NHE1 inhibitor BI‐9627 or vehicle. (B) Representative images of longitudinal sciatic nerve sections stained for CD31, F4/80, S100β, and DAPI in the indicated groups. Scale bar = 200 µm. (C) Quantification of CD31^+^ area and F4/80^+^ area in B (*n* = 5 mice). (D) Representative Western blot images showing protein levels of CD31, HIF‐1α, NHE1, p‐Dab1, Dab1, and CD68 in sciatic nerves from the indicated groups. Statistical analyses corresponding to this figure are provided in Figure S5A. (E) Schematic illustration of Dab1‐Ctrl and Dab1‐cKO mice treated with rosmarinic acid or vehicle. (F) Representative immunofluorescence images of longitudinal sciatic nerve sections from Dab1‐Ctrl and Dab1‐cKO mice treated with vehicle or rosmarinic acid, stained for CD31, F4/80, S100β, and DAPI. Scale bar = 200 µm. (G) Quantification of CD31^+^ area and F4/80^+^ area in F (*n* = 5 mice). (H) Representative Western blot images showing CD31, HIF‐1α, NHE1, p‐Dab1, Dab1, and CD68 levels in Dab1‐Ctrl and Dab1‐cKO mice treated with vehicle or rosmarinic acid. Statistical analyses corresponding to this figure are provided in Figure S5B. Data are presented as mean ± SEM. Statistical analysis: one‐way ANOVA with Tukey's post hoc test as indicated. Statistical significance was determined as indicated (^**^ *p* < 0.01, ^***^ *p* < 0.001, ^****^ *p* < 0.0001; ns, not significant).

To validate whether Dab1 regulates HIF‐1α/VEGF‐A expression and vascular regeneration via NHE1 in vivo, WT mice subjected to sciatic nerve transection received local Reelin injection with or without intraperitoneal administration of the NHE1 inhibitor BI‐9627 (10 mg/kg, every 48 h) [47] (Figure 6A). On day 5, longitudinal immunofluorescence staining showed that BI‐9627 significantly reduced CD31^+^ vessels at the lesion site and decreased the infiltration of F4/80^+^ macrophages (Figure 6B,C). Western blot analysis further showed that BI‐9627 did not alter Dab1 or p‐Dab1 levels, but reduced NHE1 expression, accompanied by marked decreases in HIF‐1α, CD31, and CD68 (Figure 6D; Figure S5A). Conversely, pharmacological activation of NHE1 by rosmarinic acid (40 mg/kg/day) [48] in Dab1‐cKO and Dab1‐Ctrl mice after sciatic nerve transection partially restored vascular and macrophage responses. On day 5, immunofluorescence staining revealed increased CD31^+^ endothelial area and F4/80^+^ macrophage signals in control mice, and a similar but attenuated increase in Dab1 cKO mice (Figure 6E–G). Western blot confirmed higher Dab1 and p‐Dab1 levels in Dab1‐Ctrl compared to Dab1‐cKO mice, with no apparent differences in Dab1 or p‐Dab1 between rosmarinic acid and vehicle treatment within the same genotype. Importantly, rosmarinic acid significantly upregulated NHE1 and increased HIF‐1α, CD31, and CD68 protein levels relative to the vehicle (Figure 6H; Figure S5B). Collectively, these in vivo data suggest that Dab1 regulates HIF‐1α expression and angiogenesis in an NHE1‐dependent manner.

### 2.7 Dab1 and Its Downstream Targets Are Activated in Human PNI Tissues

**FIGURE 7:**
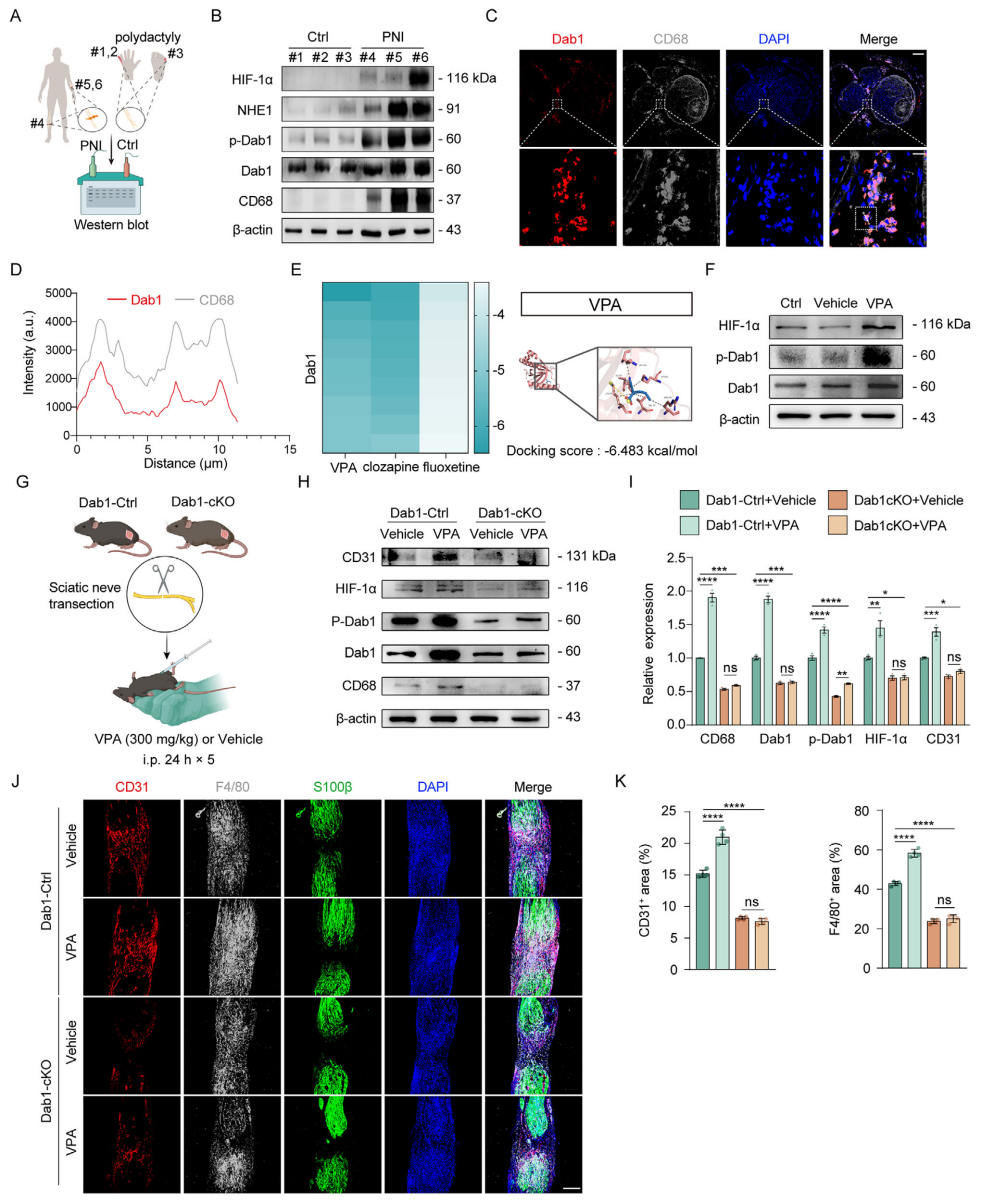
Dab1 and its downstream targets are activated in human PNI tissues. (A) Schematic showing sample collection from patients with polydactyly or with PNI, followed by protein extraction and Western blot analysis. (B) Representative Western blot images of HIF‐1α, NHE1, p‐Dab1, Dab1, and CD68 in Ctrl and PNI patient samples. Statistical analyses corresponding to this figure are provided in Figure S6A. (C) Representative immunofluorescence images of human injured median nerve sections stained for Dab1, CD68, and DAPI. Scale bar = 200 or 20 µm. (D) Line‐scan intensity profiles showing the spatial overlap of signals along the indicated regions in C. (E) Left: molecular docking analysis of VPA, clozapine, and fluoxetine with Dab1. VPA exhibits a lower binding free energy (ΔΔ*G* = −6.483 kcal/mol). Right: Molecular docking pose diagram of VPA. (F) Representative Western blot images of HIF‐1α, p‐Dab1, and Dab1 in RAW 264.7 macrophages under control, vehicle‐treated, or VPA‐treated conditions. Statistical analyses corresponding to this figure are provided in Figure S6B. (G) Schematic illustration of the in vivo experimental design. (H) Representative Western blot images showing CD31, HIF‐1α, p‐Dab1, Dab1, and CD68 in sciatic nerves from Dab1‐Ctrl and Dab1‐cKO mice treated with vehicle or VPA. (I) Quantification of the indicated proteins normalized to β‐actin in H (*n* = 3). (J) Representative immunofluorescence images of longitudinal sciatic nerve sections from Dab1‐Ctrl and Dab1‐cKO mice treated with vehicle or VPA, stained for CD31, F4/80, S100β, and DAPI. Scale bar = 200 µm. (K) Quantification of CD31^+^ vascular area and F4/80^+^ area in J (*n* = 5 mice). Data are presented as mean ± SEM. Statistical analysis: one‐way ANOVA with Tukey's post hoc test as indicated. Statistical significance was determined as indicated (^**^ *p* < 0.01, ^***^ *p* < 0.001, ^****^ *p* < 0.0001; ns, not significant).

To assess the clinical relevance of this mechanism, we analyzed surgically obtained human injured peripheral nerve samples and healthy control nerves derived from polydactyly surgeries (Figure 7A). Western blot analysis showed that, compared with healthy controls, injured nerves exhibited markedly increased levels of Dab1, p‐Dab1, NHE1, HIF‐1α, and the macrophage marker CD68 (Figure 7B; Figure S6A). Subsequently, immunofluorescence staining was performed on injured human median nerve samples, which revealed the colocalization of Dab1 with CD68 (Figure 7C,D). These data suggest that the Dab1/NHE1/HIF‐1α axis is activated in human PNI and is associated with macrophage infiltration, supporting Dab1 as a potential therapeutic target.

Based on these findings, we searched drug databases and the relevant literature for clinically translatable strategies targeting Dab1. Valproic acid (VPA), fluoxetine, and clozapine have been reported to upregulate Reelin and Dab1 expression in the rat cortex following intraperitoneal administration [49]. Computational docking analysis indicated that VPA and clozapine have strong binding affinities for Dab1, whereas fluoxetine binds only weakly (Figure 7E). Notably, VPA has also been shown to promote peripheral nerve regeneration [50, 51, 52]. Together, these observations led us to hypothesize that VPA may enhance Dab1 activity in macrophages, thereby promoting angiogenesis, alleviating hypoxia, and facilitating nerve repair.

RAW 264.7 macrophages treated with VPA for 24 h showed significantly increased Dab1, p‐Dab1, and HIF‐1α protein levels as assessed by Western blot compared with vehicle controls (Figure 7F; Figure S6B). We next tested whether VPA promotes post‐surgery angiogenesis in vivo specifically via macrophage Dab1. Sciatic nerve transection was performed in Dab1‐cKO and Dab1‐Ctrl mice, followed by daily intraperitoneal administration of VPA (300 mg/kg) [53] or vehicle until day 5 (Figure 7G). Western blot analysis of injured nerves showed that VPA increased Dab1 and p‐Dab1 in control mice, with a concomitant upregulation of HIF‐1α, CD31, and CD68. In contrast, in Dab1‐cKO mice, VPA induced only a marginal increase in p‐Dab1 (potentially from non‐macrophage sources) and did not significantly alter HIF‐1α or CD31 expression (Figure 7H,I). Consistently, immunofluorescence analysis of longitudinal sections showed that VPA significantly increased CD31^+^ endothelial area and F4/80^+^ macrophage infiltration at the injury site in control mice, but had no significant effect in Dab1‐cKO mice (Figure 7J,K).

### 2.8 Early Valproic Acid Treatment Promotes Peripheral Nerve Regeneration and Functional Recovery

**FIGURE 8:**
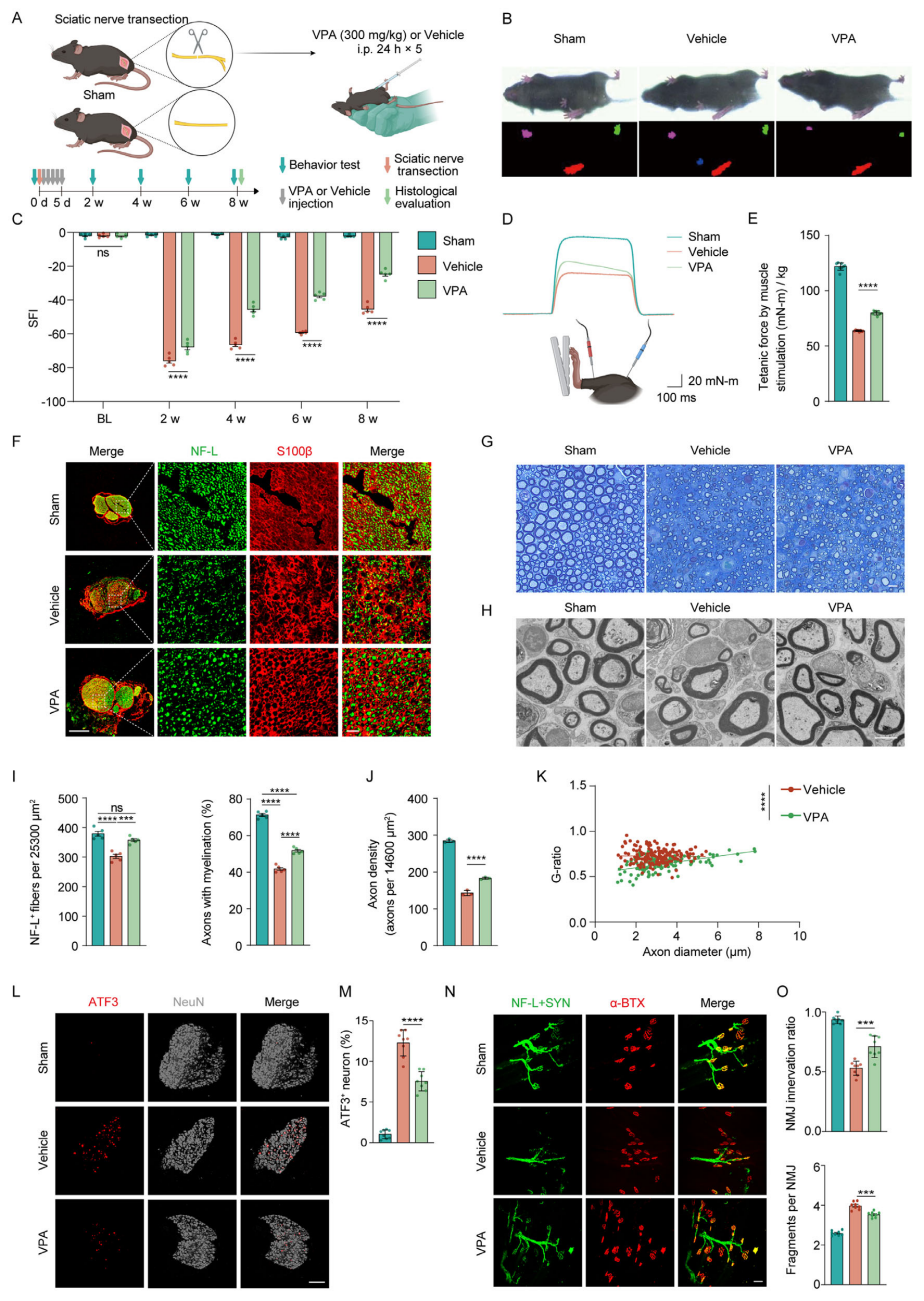
Early valproic acid treatment promotes peripheral nerve regeneration and functional recovery. (A) Schematic illustration of the experimental design and timeline. (B) Representative footprint images from sham, vehicle, and VPA‐treated groups. (C) Quantification of motor function recovery using the SFI at the indicated weeks post‐surgery (*n* = 8 mice). (D) Representative traces of tetanic force from sham, vehicle, and VPA groups. (E) Quantification of tetanic force elicited by muscle stimulation in the indicated groups (*n* = 8 mice). (F) Representative immunofluorescence images of sciatic nerve approximately 5 mm distal from injured site stained for NF‐L and S100β. Scale bars = 200 µm or 20 µm. (G) Representative toluidine blue–stained semithin sections of sciatic nerve from sham, vehicle, and VPA‐treated mice. Scale bar = 20 µm. (H) Representative TEM images demonstrating myelinated axons in the indicated groups. Scale bar = 5 µm. (I) Quantification of NF‐L^+^ fiber density and the percentage of axons with myelination in the indicated groups in F (*n* = 5 mice). (J) Quantification of axon density in sciatic nerves from the indicated groups in G (*n* = 3 mice). (K) Scatter plot of G‐ratio versus axon diameter in the indicated groups in H, only the vehicle and VPA groups are shown here for clarity. (L) Representative immunofluorescence images of DRG stained for ATF3 and NeuN. Scale bar = 200 µm. (M) Quantification of ATF3^+^ neurons in the indicated groups in L (*n* = 8 mice). (N) Representative images of NMJ stained for NF‐L/SYN and α‐BTX. Scale bar = 200 µm. (O) Quantification of NMJ innervation ratio and fragments of NMJ in the indicated groups in N (*n* = 8 mice). Data are presented as mean ± SEM. Statistical analysis: two‐way ANOVA (C) or one‐way ANOVA (E, I, J, K, M, and O) with Tukey's post hoc test as indicated. Statistical significance was determined as indicated (^***^ *p* < 0.001, ^****^ *p* < 0.0001; ns, not significant).

Based on the premise that VPA promotes peripheral nerve regeneration by enhancing angiogenesis, we hypothesized that administering VPA during the critical vascular‐regeneration window (days 1–5 post‐injury) would improve axonal regeneration and functional recovery. To test this, a sciatic nerve transection model was established in WT mice, which received daily intraperitoneal administration of VPA until postoperative day 5 (Figure 8A). Gait was assessed every 2 weeks using the DigiGait imaging system. At 8 weeks, thermal imaging showed that VPA increased the maximal plantar contact area compared with the vehicle‐treated group, although values remained slightly below those of the sham group (Figure 8B). SFI analysis showed significant improvement in the VPA group beginning at 2 weeks, which remained consistently superior to the vehicle group throughout the follow‐up period, without fully reaching sham levels (Figure 8C). Consistently, tibialis anterior strength testing at 8 weeks demonstrated a greater recovery of contractile force in the VPA group than in the vehicle controls (Figure 8D,E).

Nerve regeneration was evaluated approximately 5 mm distal to the lesion site. Immunostaining, toluidine blue staining, and transmission electron microscopy (Figure 8F–H) collectively demonstrated that VPA‐treated nerves exhibited a significantly higher number of NF‐L^+^ fibers and a marked increase in remyelinated axons. Quantitative analysis further revealed increased axon density and a reduced G‐ratio in the VPA group (Figure 8I–K; Figure S7A–C), indicating enhanced axonal regeneration and remyelination. In addition, we assessed the DRG corresponding to the injured segments, as well as neuromuscular junction (NMJ) reinnervation in the extensor digitorum longus muscle. VPA treatment reduced the number of ATF3^+^ neurons at 8 weeks (Figure 8L,M), suggesting an attenuation of injury‐induced neuronal stress responses [54]. Moreover, NMJ staining showed increased innervation of acetylcholine receptor clusters and decreased receptor fragmentation in VPA‐treated mice (Figure 8N,O), supporting improved terminal reinnervation [55, 56]. Together, these findings indicate that VPA administered during the vascular‐regeneration window enhances angiogenesis via the Dab1/NHE1/HIF‐1α axis, thereby promoting axonal regeneration and functional recovery.

## 3 Discussion

Hypoxia is a key early event in the injury microenvironment following PNI. Previous studies have shown that pronounced endoneurial hypoxia can be detected at the injury site as early as approximately 2 h [30]. Most prior research has investigated nerve repair outcomes from a single‐intervention perspective, either focusing on hypoxia response pathways or on improving oxygen supply [3, 12]. However, the dynamic temporal evolution and spatial distribution patterns of hypoxia after nerve injury have not been systematically characterized, and its causal role and critical time window remain poorly defined. To address this gap, we systematically delineated the spatiotemporal dynamics of hypoxia following PNI and further found that, although exogenous oxygen supplementation reduced the number of hypoxic cells at early stages (day 5), it paradoxically inhibited nerve regeneration in the long term. Mechanistically, exogenous oxygen delivery attenuated HIF‐1α‐mediated hypoxic responses, thereby suppressing early angiogenesis after injury. Early vascular reconstruction is considered a crucial prerequisite for the initiation of regeneration, as it not only provides metabolic support to the local tissue, but also serves as a structural scaffold that guides the directed migration of repair Schwann cells and the formation of Büngner bands, thus creating continuous growth conduits for regenerating axons [5, 57]. Therefore, our findings emphasize that a strategy more consistent with physiological repair processes is not to directly “counteract hypoxia” during the early phase after injury. Instead, hypoxia‐triggered vascular remodeling signals should be harnessed and amplified within an appropriate therapeutic window (1–5 days post‐injury), prioritizing activation of the HIF‐1α pathway and enhancing the expression and secretion of pro‐angiogenic factors such as VEGF‐A.

Recently, Dab1 has been increasingly recognized for functions beyond its classical roles. Marta Segarra and colleagues reported that endothelial Dab1 promotes coordinated central angiogenesis during brain development, facilitating vascular–glial–neuronal interactions and establishing blood–brain barrier integrity [19]. While Dab1 has been extensively studied in neural and vascular development, its functions in immune cells remain relatively unexplored. Previous studies have shown that ApoER2‐associated stimulation in monocyte‐like cells (U937) can induce rapid Dab1 phosphorylation and activate signaling pathways such as PI3K/Akt [58], suggesting that Dab1 can be recruited and participate in signal transduction in myeloid cells. However, its biological roles and underlying mechanisms in the context of injury repair have remained largely uncharacterized. Here, we demonstrate that Dab1 is markedly upregulated following PNI and is predominantly localized in macrophages, where it plays a critical role in hypoxia‐driven vascular reconstruction. Notably, Dab1 activation also induced a pronounced morphological polarization in these cells. Given the intrinsic link between macrophage polarization and their functional phenotype, our findings suggest that Dab1 may regulate not only angiogenesis but also canonical macrophage functions, such as phagocytosis and inflammatory mediator secretion [59, 60].

Following PNI, the local hypoxic microenvironment serves as an upstream trigger to activate Dab1 signaling in macrophages. This hypoxia‐driven activation, in turn, results in their robust recruitment and infiltration into the lesion site. It has been reported that NIK/NF‐κB pathway activation can upregulate chemokine receptors and adhesion molecules, molecularly priming macrophages to recognize and adhere to the injured nerve [61]. Concurrently, calcium‐mediated signaling can drive rapid cytoskeletal remodeling and actomyosin contraction, providing the mechanical motility required for macrophages to migrate into the lesion [62, 63]. These pathways are essential for enhancing the chemotactic sensitivity of macrophages to early injury signals and for driving the rapid cytoskeletal dynamics required for active migration. Intriguingly, our GO enrichment analysis of the proteomic data reveals the enrichment of both NIK/NF‐κB and calcium‐mediated signaling pathways, suggesting that hypoxia‐activated Dab1 may act as a molecular hub coordinating these pathways to regulate macrophage infiltration.

Specifically, macrophage‐specific Dab1 deletion led to reduced HIF‐1α/VEGF‐A expression and decreased regenerative angiogenesis, whereas activation of Dab1 by Reelin enhanced HIF‐1α and VEGF‐A expression and promoted angiogenesis. These findings support Dab1 as an important upstream regulator of HIF‐1α signaling in macrophages. Further mechanistic investigation revealed that NHE1 participates in Dab1‐mediated regulation of HIF‐1α, a finding consistent with previous reports showing that NHE1 contributes to HIF‐1α–associated angiogenic programs [46]. Notably, NHE1 activation alone was insufficient to fully restore HIF‐1α expression and angiogenic phenotypes in the absence of Dab1, suggesting that Dab1 regulates the HIF‐1α axis through multiple, parallel pathways rather than a single NHE1‐dependent mechanism. In addition to modulating intracellular pH, Dab1 activates the PI3K/Akt pathway, which enhances HIF‐1α protein levels and transcriptional activity through mTOR‐dependent translational control and, in certain contexts, protein stabilization [58, 64]. We therefore propose that Dab1 indirectly regulates NHE1 through PI3K/Akt/mTOR signaling, promoting NHE1 phosphorylation, membrane localization, and exchanger activity, leading to intracellular alkalinization and coordinated activation of hypoxia‐responsive gene expression [65]. Through this integrated Dab1/PI3K/Akt/mTOR/NHE1 axis, Dab1 signaling may link extracellular cues to pH homeostasis and hypoxia‐driven adaptation during tissue regeneration.

Classically, Reelin stimulates the phosphorylation of Dab1 to amplify downstream signaling. However, we observed an unexpected and delayed increase in *Dab1* mRNA and total Dab1 protein levels at 60 min and 24 h. Because existing literature indicates that Reelin controls Dab1 strictly through a post‐translational negative feedback loop—rather than via direct transcriptional regulation–we hypothesize that several context‐specific mechanisms may drive this accumulation [66]. First, the early Reelin‐induced Dab1 activation might initiate an indirect positive feedback loop, where phosphorylated Dab1 stimulates downstream cascades such as the Akt/mTOR or MAPK/ERK pathways to promote its own protein translation or stability [67, 68]. Given the established role of HIF‐1α as a master transcriptional regulator under hypoxic conditions, it is conceivable that HIF‐1α may participate in the regulation of Dab1 expression through yet unidentified transcriptional or epigenetic mechanisms [69, 70]. Last, it is also highly possible that macrophage‐specific variations in protein turnover, such as a deficiency in the Cul5‐dependent degradation machinery or the recruitment of specific deubiquitinating enzymes, might shield phosphorylated Dab1 from proteasomal degradation and permit its progressive cellular accumulation [71, 72].

As a clinically long‐used mood stabilizer, VPA exerts pleiotropic effects beyond neuroelectrophysiological modulation and neurotransmitter regulation [73, 74], with well‐characterized epigenetic regulatory properties. VPA inhibits histone deacetylases, thereby modulating chromatin accessibility and transcriptional activity to regulate gene programs involved in tissue injury and repair [75]. A number of animal studies have reported that VPA improves both morphological and functional outcomes in sciatic nerve injury models [50, 51]; however, the specific biological processes and key targets implicated have not been fully consistent, suggesting that VPA regulates regeneration through multiple convergent pathways. Our study identifies a previously unrecognized mechanism centered on early injury microenvironment remodeling rather than neuron‐ or Schwann cell‐intrinsic effects. We show that VPA acts on macrophages during the early post‐injury phase to activate Dab1 and amplify HIF‐1α signaling, thereby increasing the expression and secretion of pro‐angiogenic factors such as VEGF‐A and accelerating vascular reconstruction. Notably, this pro‐angiogenic effect is abolished in macrophage‐specific Dab1 deficiency, indicating that the early therapeutic action of VPA depends on a defined cell type and molecular target. Consistent with prior reports demonstrating the immunomodulatory effects of VPA on macrophage phenotypes [76, 77], our findings establish macrophage Dab1 as a critical regulatory node connecting VPA treatment with angiogenic remodeling after peripheral nerve injury. Intriguingly, we also observed that VPA increased Dab1 phosphorylation in macrophage Dab1‐cKO mice, which might be the compensatory activation of Dab1 in endothelial cells. Previous studies have shown that activation of endothelial Dab1 in the central nervous system can promote vascular formation [19], however, we did not observe a corresponding increase in CD31^+^ vessels. We propose two possible explanations for this. First, the limited activation of Dab1 in endothelial cells at this stage might not be sufficient to drive significant angiogenesis. More importantly, the deletion of Dab1 in macrophages markedly reduces VEGF‐A secretion (Figure S2D), which likely impairs upstream angiogenic signaling and thereby restricts the ability of endothelial Dab1 to promote vascular regeneration.

In summary, our findings demonstrate that hypoxic responses after PNI are highly time‐dependent and stage‐specific. Rather than being entirely harmful, early hypoxia acts as an internal signal that triggers repair processes. Based on this concept, we propose a therapeutic strategy that goes beyond simply eliminating hypoxia. Instead, it uses early hypoxic signals within a critical time window to selectively promote blood vessel repair via macrophage Dab1 and its downstream signaling. By targeting vascular recovery, hypoxic stress can be relieved at its source, thereby promoting overall regeneration. This strategy—adapting to hypoxia and prioritizing blood vessels—provides a new framework for understanding the link between the vascular system, immune response, and regeneration after PNI. Furthermore, it offers a precisely timed approach for future targeted treatments.

## 4 Materials and Methods

### 4.1 Patient Samples

Human peripheral nerve samples were obtained from the Department of Hand and Foot Surgery, China‐Japan Union Hospital of Jilin University. The use of human tissues in this study was approved by the Ethics Committee of China‐Japan Union Hospital of Jilin University (approval no. 2025071603). Written informed consent was obtained from all participants or their legal guardians before tissue collection and inclusion in the study. Patients with uncontrolled hypertension or metabolic disorders were excluded, as these chronic conditions may independently contribute to neurodegenerative changes. Peripheral nerve samples were collected from discarded nerve tissues excised during surgery from patients with severe peripheral nerve injury, as well as from digital nerves obtained during digit amputation in patients with congenital polydactyly. Residual nerve segments (approximately 2 cm) from amputated limbs were rapidly snap‐frozen on dry ice to minimize injury‐induced artifacts. These samples were used for protein extraction and subsequent Western blot analysis according to established protocols.

### 4.2 Animals

All animal procedures were approved by the Animal Ethics and Welfare Committee of Jilin University (approval no. 2024328). Mice were housed at a maximum of five animals per cage under a 12 h light/dark cycle, with free access to water and standard rodent chow (Diet 7097, Harlan Teklad). Ambient temperature was maintained at ∼23°C. All genetically modified mice used in this study were on a C57BL/6 background. Dab1‐flox mice (Dab1^fl/fl^) and Lyz2‐Cre^ERT2^ mice (C001499) were obtained from Cyagen (Suzhou, China). Wild‐type male C57BL/6 mice were purchased from Changsheng Biotechnology.

### 4.3 Genotyping

Genotyping was performed via PCR amplification of genomic DNA. DNA was isolated from the toes of young mice within 7 days after birth by incubation in 75 µL Lysis Buffer containing 25 mm NaOH and 0.2 mm EDTA for at least 1 h at 98°C under constant shaking. Then the temperature of the mixture was reduced to 15°C, and the same volume of buffer (40 mm Tris‐HCl, pH 5.5) was added. The liquid was mixed and used for subsequent PCR assays [78].

### 4.4 Tamoxifen Injection

For inducible macrophage‐specific Dab1 knockout. Dab1^fl/fl^; Lyz2‐Cre^ERT2^ and their littermate control (Dab1^fl/fl^) mice received i.p. injections of tamoxifen (100 mg/kg) dissolved in corn oil and ethanol every other day for three days [78].

### 4.5 Sciatic Nerve Transection

All experiments were performed in adult male C57BL/6 mice (8 weeks old, 18–22 g). Animals were randomly assigned to experimental groups. Mice were anesthetized with isoflurane (5% for induction and 2% for maintenance), and the surgical area (hind limbs and lower back) was shaved and disinfected with iodine. Ophthalmic ointment was applied to prevent corneal drying. A skin incision was made over the mid‐thigh, and the biceps femoris and gluteus superficialis muscles were bluntly separated to expose the sciatic nerve. The sciatic nerve was transected approximately 1 cm proximal to its bifurcation, and both the proximal and distal stumps were fixed in situ with 10‐0 sutures positioned 0.5 cm from the transection site [5, 79, 80]. The incision was sutured, and animals were monitored closely until they woke up completely and were able to eat and drink properly. Pain management was conducted by adding Metamizole (500 mg per 100 mL drinking water) from 1 day before the surgery until 3 days post‐surgery.

### 4.6 Local Application of Calcium Peroxide (CaO_2_)

Sterile CaO_2_ nanoparticles were embedded in a thermosensitive Pluronic F‐127 hydrogel vehicle (20% w/v in PBS) to obtain a working suspension of 2 mg/mL. For local topical application, 10 µL of the CaO_2_ suspension (equivalent to 20 µg CaO_2_ per nerve) was pipetted directly onto the epineurium of the exposed sciatic nerve prior to wound closure [26, 27, 28]. Vehicle control animals received Pluronic F127 hydrogel with Ca(OH)_2_.

### 4.7 Local Application of Reelin

Reelin was diluted in sterile PBS containing 0.1% BSA to the desired working concentration and kept on ice. A dose of 10 µg per nerve in 10 µL was used [81]. Using a 10 µL Hamilton syringe fitted with a 27 G needle, 10 µL of Reelin solution was slowly injected into the perineural space adjacent to the epineurium, taking care not to penetrate the nerve fascicle (injection rate: 1 µL per 5–10 s). After injection, the needle was left in place for 10–20 s and then slowly withdrawn to minimize backflow. Vehicle control animals received 0.1% BSA in PBS.

### 4.8 BI‐9627 Preparation and Administration

BI‐9627 was first dissolved in DMSO to prepare a 1 mg/mL stock solution, and then diluted 1:9 with sterile corn oil to obtain the final dosing solution. Mice received BI‐9627 at 10 mg/kg body weight by intraperitoneal injection every other day. The injection volume was 10 mL/kg. Vehicle control animals received 10% DMSO/90% corn oil without BI‐9627 [47]. All formulations were prepared immediately before administration and protected from light. Injections were performed using sterile syringes fitted with 27 G needles.

### 4.9 Rosmarinic Acid Preparation and Administration

Rosmarinic acid was dissolved in a composite vehicle consisting of 10% dimethyl sulfoxide (DMSO), 40% PEG300, 5% Tween‐80, and 45% normal saline. After sciatic nerve transection, the solution was administered daily via intraperitoneal injection at 40 mg/kg until postoperative day 5. Control animals received an equal volume of vehicle [82, 83].

### 4.10 VPA Preparation and Administration

VPA was dissolved in PBS. VPA was administered once daily at a dose of 300 mg/kg via intraperitoneal injection after sciatic nerve transection, continuing until the fifth day post‐surgery [50]. Control animals received an equal volume of PBS.

### 4.11 Detection of Hypoxic Tissues Using the Hypoxyprobe Kit

Forty‐five min before tissue collection, mice were intravenously injected via the tail vein with pimonidazole (60 mg/kg). Tissues were then harvested, fixed, and processed to prepare frozen sections [22, 24]. Hypoxic regions were detected by immunohistochemical staining using an anti‐pimonidazole antibody according to standard protocols.

### 4.12 DigiGait Gait Analysis

Mice were placed on a transparent motorized treadmill set at a constant speed of 20 cm/s. A high‐speed camera was installed beneath the treadmill to record paw movement trajectories in real time during locomotion. Prior to baseline testing, mice underwent acclimation training for three consecutive days to ensure stable walking performance, with each session lasting no less than 6 s. After acclimation training, mice were allowed to rest for 1 day before surgical procedures. During testing, data recording was performed only when mice were able to walk continuously and steadily at 20 cm/s for more than 5 s [84]. The DigiGait software was used to automatically calculate the SFI, enabling quantitative assessment of gait function and evaluation of nerve recovery.

### 4.13 Tetanic Force Measurement

Mice were anesthetized and placed on a 37°C heating pad. The left foot was secured to the foot plate attached to the instrument (Aurora Scientific, 1300A), while the left knee was gently stabilized with the knee clips to ensure its stability. The angle of the foot plate was set at a default value of 17° but could be adjusted for optimal twitch force. Muscle stimulation was achieved through direct stimulation using two needle‐like electrodes with a current intensity of 2 mA. The stimulus pulse width remained constant at 0.2 ms throughout all experiments. Tetanic contractions were induced by delivering stimuli lasting 300 ms at frequencies of 50, 100, and 125 Hz, respectively. A resting interval of 2 min separated each tetanic contraction session. Tetanic contraction forces were normalized based on body weight [85, 86].

### 4.14 Immunofluorescence Staining

Mice were deeply anesthetized with isoflurane and perfused with PBS followed by 4% paraformaldehyde (PFA). For sciatic nerve and dorsal root ganglion (DRG) staining, tissues were dissected, fixed in 4% PFA at 4°C overnight, and cryoprotected in 30% sucrose at 4°C for 24–48 h. Sections (12 µm) were prepared using a cryostat microtome (Leica, CM1950). Sections were washed with PBS, blocked in 5% donkey or goat serum containing 1% Triton X‐100 for 2 h at room temperature, and incubated with primary antibodies overnight at 4°C. After washing with PBST (0.5% Tween‐20), sections were incubated with fluorophore‐conjugated secondary antibodies for 2 h at room temperature. Samples were then washed, coverslipped, and imaged using a laser‐scanning confocal microscope (Nikon, A1R).

### 4.15 Neuromuscular Junction Staining

As previously described [78], the extensor digitorum longus muscle was isolated and fixed in 4% PFA at 4°C overnight, then washed in 0.1 m glycine in PBS for 30 min. Samples were blocked in buffer containing 2% Triton X‐100, 5% BSA, and 5% goat serum for 2 h at room temperature. After blocking, muscles were incubated with primary antibodies overnight at 4°C. Following three washes, samples were incubated with secondary antibodies overnight at 4°C. Images were acquired using a laser‐scanning confocal microscope (Nikon, A1R). Quantification included analysis of fragmented endplates and mono‐innervated junctions.

### 4.16 Toluidine Blue Staining

To evaluate the morphological changes of myelinated nerve fibers, mice were euthanized, and sciatic nerves were excised and fixed in 2.5% glutaraldehyde in 0.1 m PBS at 4°C overnight. The samples were then post‐fixed in 1% osmium tetroxide for 1 h, dehydrated through a graded ethanol series (50%, 70%, 80%, 90%, 95%, and 100%), cleared in propylene oxide, and embedded in epoxy resin. Semi‐thin transverse sections of approximately 1 µm thickness were obtained using an ultramicrotome (LEICA, EM UC7) and mounted on glass slides. The sections were stained with 1% toluidine blue in 1% sodium borate at 60°C for 1–2 min, rinsed thoroughly with distilled water, air‐dried, and mounted with neutral balsam [87]. The stained sections were examined under a light microscope (LEICA, DFC7000T), and images were captured for morphometric analysis of myelinated fiber density.

### 4.17 Transmission Electron Microscopy (TEM)

For ultrastructural analysis, mice were euthanized, and sciatic nerves were excised and fixed in 2.5% glutaraldehyde at 4°C overnight, followed by post‐fixation in 1% osmium tetroxide [88]. Samples were rinsed in phosphate buffer, dehydrated through a graded ethanol series, and embedded in epoxy resin. Ultrathin sections were prepared using an ultramicrotome (LEICA, EM UC7), mounted onto copper grids, and stained with 1% uranyl acetate and lead citrate. Images were acquired using a JEOL transmission electron microscope. Myelin sheath thickness was quantified using the g‐ratio (axon diameter/fiber diameter).

### 4.18 Ex Vivo Sciatic Nerve Explant Culture

Sciatic nerves were isolated from adult mice, and the epineurium and perineurium were carefully removed under a stereomicroscope using fine microforceps [31, 89]. Nerve explants were cultured in 2 mL of ex vivo medium consisting of DMEM/F12 supplemented with 0.5 mm pyruvate, 1× GlutaMAX, 1× penicillin/streptomycin, and 1× N2 supplement. Cultures were maintained at 37°C in a humidified incubator containing 5% CO_2_. Experimental groups were exposed to hypoxia for 0, 1, 3, or 5 days. At the indicated time points, explants were collected for immunofluorescence staining.

### 4.19 Cell Cultures

Murine macrophages RAW 264.7 cells (RRID: CVCL_0493) were purchased from Procell Life Science & Technology (Wuhan, China; Cat. No. CL‐0190). Human umbilical vein endothelial cells (HUVECs; RRID: CVCL_2959) were purchased from Zhejiang Meisen Cell Technology (Zhejiang, China; Cat. No. CTCC‐009‐493). Cell line authentication and verification of mycoplasma‐free status were conducted by the respective suppliers prior to distribution. The cells were cultured according to the suppliers' recommended protocols: RAW 264.7 cells were maintained in DMEM, and HUVECs were cultured in ECM. Both media were supplemented with 10% FBS, and the cells were incubated at 37°C in a humidified atmosphere containing 5% CO_2_.

### 4.20 siRNA Interference

For RNA interference, Dab1 siRNA, NHE1 siRNA, and negative control siRNA (Integrated Biotech Solutions) were transfected using Lipofectamine 3000 reagent [89]. After 6 h of transfection, the medium was replaced with fresh DMEM medium containing 10% FBS for additional culture of 24 h prior to downstream analysis. The negative control was designed as a nonspecific sequence.

### 4.21 Cell Immunofluorescence Staining

RAW 264.7 macrophages were seeded on glass coverslips and fixed with 4% PFA for 15 min at room temperature. Cells were then permeabilized with 0.2% Triton X‐100 in PBS for 10 min and blocked with 5% BSA for 1 h to minimize nonspecific binding. Subsequently, cells were incubated overnight at 4°C with primary antibodies. The secondary antibodies were incubated 2 h at room temperature; cells were washed and mounted using coverslips. Images were acquired using a laser‐scanning confocal microscope (Nikon, A1R).

### 4.22 Concentration of Cell Supernatant

The culture supernatant of RAW 264.7 macrophages was collected and centrifuged at 1000 ×* g* for 10 min to remove cell debris. The clarified supernatant was then concentrated using a 30 kDa molecular weight cut‐off centrifugal filter unit according to the manufacturer's instructions. Briefly, the supernatant was loaded into the ultrafiltration device and centrifuged at 4000 × *g* at 4°C until the volume was reduced to approximately 1/10 of the original volume. The retentate was collected and stored at −80°C for further analysis.

### 4.23 ELISA Analysis

VEGF‐A levels in mouse tissues and cultured cell supernatants were quantified using a commercial VEGF‐A ELISA kit (MM‐44452M1, MEIMIAN) according to the manufacturer's instructions. Briefly, tissue homogenates and cell supernatants were added to antibody‐coated 96‐well plates, followed by incubation with detection antibody and HRP‐conjugated secondary reagent. After washing, TMB substrate was added for color development, and the reaction was terminated with stop solution. Absorbance was measured at 450 nm using a microplate reader. VEGF‐A concentrations were calculated from a standard curve.

### 4.24 5‐Ethynyl‐2′‐Deoxyuridine (EdU) Test

Cell proliferation was assessed using an Apollo EdU Imaging Kit following the manufacturer's protocol. Briefly, cells were incubated with 10 µm EdU for 2 h at 37°C, washed with PBS, fixed with 4% paraformaldehyde for 15 min, and permeabilized with 0.2% Triton X‐100 for 10 min. The click reaction was performed for 30 min at room temperature using an Alexa Fluor 567‐conjugated azide. Nuclei were counterstained with Hoechst, and images were captured by fluorescence microscopy. The proliferation index was calculated as the percentage of EdU^+^ nuclei over total Hoechst‐stained nuclei [90]. Images were acquired using a laser‐scanning confocal microscope (Nikon, A1R). At least three independent experiments with ≥5 fields per sample were analyzed.

### 4.25 Tube Formation Assay

To evaluate endothelial angiogenic capacity in vitro, growth factor‐reduced Matrigel was thawed at 4°C overnight and added (150 µL per well) to prechilled 24‐well plates, followed by incubation at 37°C for 1 h to allow polymerization. HUVECs were harvested, resuspended in complete endothelial growth medium, and seeded onto the solidified Matrigel at a density of 1.5 × 10^5^ cells per well. Cells were then treated with the indicated agents and incubated at 37°C in a humidified incubator containing 5% CO_2_. After 4 h, tube formation was observed under an inverted phase‐contrast microscope (Nikon, DS‐Ri2), and representative images were captured from at least three random fields per well. Quantitative analysis of capillary‐like networks was performed using ImageJ with the Angiogenesis Analyzer plugin. Total tube length, number of junctions, and number of segments were quantified [91].

### 4.26 Transwell Analysis

RAW 264.7 macrophages were serum‐starved for 6 h prior to the assay. Reelin (0.1 µg/mL) or PBS was added for pretreatment of 12 h. Cells were resuspended in serum‐free medium, and approximately 1 × 10^5^ cells in 200 µL were seeded into the upper chamber. The lower chamber was filled with 600 µL of medium containing 10% FBS as a chemoattractant. Plates were incubated at 37°C, 5% CO_2_. After 24 h, the medium was removed, and the transwell chamber was fixed with 4% PFA at room temperature for 10 min. It was then washed 1–2 times with PBS, stained with 0.1% crystal violet for 8 min, and the staining solution was discarded. After washing 1–2 times, the upper layer of the chambers was gently wiped, and the positive cells were observed and counted under a microscope (Nikon, DS‐Ri2). Five or ten images from each group were selected and independently assessed by unbiased observers to represent the migration ability of RAW 264.7 macrophages under different conditions. For the HUVECs were serum‐starved for 6 h prior to the assay. Cells were resuspended in medium, and approximately 1 × 10^5^ cells in 200 µL were seeded into the upper chamber. The lower chamber was filled with 600 µL of medium containing 10% RAW 264.7 retentate as a chemoattractant. The following steps were the same as in the previous experiment [92].

### 4.27 Real‐Time qPCR Analysis

Total RNA was extracted using the Eastep Super Total RNA Extraction Kit (Promega, LS1040). cDNA was synthesized using Transcript One‐Step cDNA Synthesis SuperMix (TransGen Biotech, AT311). Primers were synthesized by Genewiz Biotech as listed in Table S3. cDNA templates and primers were combined with TB Green Premix Ex Taq (TaKaRa, RR420A) for quantitative real‐time PCR using a Bio‐Rad CFX96 real‐time PCR system. Relative expression levels were calculated using the 2^−ΔΔ^ *^Ct^* method. β‐Actin was used as the internal control.

### 4.28 Western Blot Analysis

Primary Schwann cells or sciatic nerves were lysed in RIPA buffer supplemented with 1× protease inhibitor cocktail. Protein concentrations were determined using a BCA assay. Equal amounts of protein were separated by 8%–12% SDS‐PAGE and transferred onto PVDF membranes. Membranes were blocked for 1.5 h at room temperature and incubated with primary antibodies overnight at 4°C. Membranes were then incubated with secondary antibodies for 2 h at room temperature. After signal detection, excess developing solution was washed off with TBST, and membranes were incubated in western blot antibody stripping solution at room temperature for 20 min to remove bound antibodies. Membranes were then washed with TBST, re‐blocked, and reprobed as described above. Images were scanned using a GS800 densitometer and analyzed with PDQuest 7.2.0 software. Band intensities were normalized to loading controls. Experiments were repeated three times, and representative images are shown.

### 4.29 Protein Digestion and TMT Labeling

Samples were washed with PBS in an Eppendorf (EP) tube, followed by the addition of protease and trypsin for digestion. The resulting tryptic peptides were then transferred to a 1.5 mL tube to terminate the digestion process. After confirming the pH of the samples, desalting was performed using the SOLAµ system (Thermo Fisher Scientific). Subsequently, the peptides were labeled with the TMTpro 16‐plex Isobaric Label Reagent Set. The peptides were separated using high‐performance liquid chromatography (HPLC) and combined into 30 fractions. After that, samples were sent for LC‐MS analysis. The mass spectrometry proteomics data generated in this study were deposited in PRIDE under project accession PXD081372.

### 4.30 Molecular Docking Analysis

First, AutoDockTools software (Version 1.5.7) was used to preprocess the small molecules and proteins, select appropriate docking sites, and export configuration files for AutoDock Vina docking. Next, AutoDock Vina was employed to perform continuous local searches and iterative optimization to identify the optimal molecular docking conformations. Finally, PyMOL (Version 3.0.3) and LigPlot (Version 2.2.9) were used for result analysis and visualization.

### 4.31 Statistical Analysis

All data are shown as mean ± SEM. Sample size was determined based on previous experiments using similar methodologies. Data were analyzed by GraphPad Prism 10.0. Statistical comparisons were made using a two‐tailed *t*‐test, one‐way ANOVA, or two‐way ANOVA as indicated in the figure legends. Differences were considered significant when *p *< 0.05. Asterisks correspond to the following significance levels: ns, not significant, *p < 0.05, **p < 0.01, ***p < 0.001, and ****p < 0.0001.

## Author Contributions

Concept and design: Xiongyao Zhou, Fan Zhang, and Rangjuan Cao. Acquisition, analysis, and interpretation of the data: Xiongyao Zhou, Jiangbo Wang, Huiyuan Wang, Zongqiang Wang, Han Wang, Qianqian Wang, Le Qi, and Ziyi Zhao. Drafting the manuscript: Xiongyao Zhou, Chuikai Meng, Jialu Sun, and Weiye Li. Illustrations were created by Xiongyao Zhou using BioRender. Statistical analysis: Xiongyao Zhou, Fan Zhang, and Jiangbo Wang. Administrative, technical, or material support: Huiyuan Wang, Xiaosong Gu, Shuseng Cui, and Rangjuan Cao.

## Funding

This research was supported by National Natural Science Foundation of China (82471410, 82271414); Jilin Province Special Project for Health Research Talents (2023SCZ67, 2023SCZ07); Foreign Expert Program (D20240202); Jilin Provincial Budgeted Capital Construction Fund (Innovation Capacity Building) Project (2024C011‐6); Young Top Talent Project of the Jilin Provincial Changbai Talents Program (202441127); Jilin Province Science and technology innovation platform construction project (YDZJ202602CXJD076); Norman Bethune Program of Jilin University (2022B07).

## Conflicts of Interest

The authors declare no conflicts of interest.

## Supporting information




**Supporting File**: advs77705‐sup‐0001‐SuppMat.docx.

## Data Availability

The data and materials that support the findings of this study are available from the corresponding author upon reasonable request.
