# Supplementary material for: Macrophage Dab1 Links Early Hypoxia to Adaptive Angiogenesis to Drive Peripheral Nerve Repair
Source: Adv Sci (Weinh). 2026 Sep 10:e77705. Online ahead of print. doi: 10.1002/advs.77705 (PMC13562779; doi:10.1002/advs.77705)
Supplement: Supplementary file 1 [file ADVS-9999-e77705-s001.docx]

**Supporting Information**

**Figures S1-S7**

**Figure S1.** **Dab1 is a hypoxia-responsive protein following PNI.** **(A)** GEO dataset (GSE45376) analysis. Volcano plot showing differentially expressed genes between WT mice at 0 and 7 days after SCI. DEGs were defined as genes with *P* < 0.05 and |log_2_ fold change| > 1. **(B)** Representative immunofluorescence images of sciatic nerve cross-sections stained for Dab1, F4/80, NF-200, and DAPI. Scale bars = 200 μm or 20 μm. **(C)** Line-scan intensity profiles showing the spatial overlap of Dab1 with F4/80 and NF-200 along the indicated regions. **(D)** Representative immunofluorescence images of sciatic nerve cross-sections stained for Dab1, CD31, S100β and DAPI. Scale bars = 200 μm or 20 μm. **(E)** Line-scan intensity profiles showing the spatial overlap of Dab1 with CD31 and S100β along the indicated regions.

**Figure S2. Macrophage Dab1 mediates hypoxia-induced HIF-1α/VEGF-A expression and angiogenesis.** **(A)** Representative immunofluorescence images of sciatic nerve cross-sections stained for Dab1, F4/80, and DAPI in Dab1-Ctrl or Dab1-cKO groups. Scale bars = 200 μm or 20 μm. **(B)** Line-scan intensity profiles showing the spatial overlap of Dab1 with F4/80 along the indicated regions. **(C)** Quantification of relative expression of CD31, HIF-1α, p-Dab1, Dab1, and CD68 in Figure 3D (n = 3). **(D)** ELISA analysis of VEGF-A protein levels of sciatic nerve from Dab1-Ctrl and Dab1-cKO mice. **(E)** Quantification of relative expression of CD31, HIF-1α, p-Dab1, Dab1, and CD68 in Figure 3F (n = 3). **(F)** Representative immunofluorescence images of DRG stained for ATF3 and NeuN. Scale bar = 200 μm. **(G)** Quantification of ATF3⁺ neurons in indicated groups in F (n = 5 mice). Data are presented as mean ± SEM. Statistical analysis: one-way ANOVA with Tukey’s post hoc test (E and G) and unpaired two-tailed Student’s t-test (C and D), as indicated. Statistical significance was determined as indicated (^**^*P* < 0.01, ^***^*P* < 0.001, ^****^*P* < 0.0001; ns, not significant).

**Figure S3. Activation of macrophage Dab1 orchestrates a hypoxia-like angiogenic response via the HIF-1α/VEGF-A axis.** **(A)** RT-qPCR analysis of *Dab1* and *VEGF-A* mRNA expression in RAW 264.7 macrophages treated with vehicle or increasing concentrations of Reelin (n = 4). **(B)** Representative Western blot images of HIF-1α, p-Dab1 and Dab1 in RAW 264.7 macrophages treated with Reelin for the indicated time points, with β-actin as a loading control. **(C)** Quantification of relative expression of HIF-1α, p-Dab1 and Dab1 in B (n = 3). **(D)** RT-qPCR analysis of *Dab1* mRNA expression in RAW 264.7 macrophages treated with Reelin for the indicated durations (n = 4). **(E)** Representative images of migrated HUVECs under Ctrl, vehicle, or Reelin treatment. Scale bar = 20 μm. **(F)** Quantification of migrated cells in E (n = 5). **(G)** Representative immunofluorescence images of HUVECs stained for EdU and DAPI. Scale bar = 20 μm. **(H)** Quantification of the EdU^+^ cell ratio in G (n = 5). **(I)** Representative images of migrated HUVECs following Ctrl, si-Ctrl, and si-Dab1–transfected RAW 264.7 cells. Scale bar = 20 μm. **(J)** Quantification of migrated cells in I (n = 5). **(K)** Representative immunofluorescence images of HUVECs stained for EdU and DAPI. Scale bar = 20 μm. **(L)** Quantification of the EdU^+^ cell ratio in K (n = 5). **(M)** Representative images of migrated RAW 264.7 cells in response to Ctrl, vehicle, and Reelin treatment. Scale bar = 20 μm. **(N)** Quantification of migrated cells in M (n = 10). **(O)** Representative EdU staining of RAW 264.7 cells treated with Ctrl, vehicle, or Reelin. Scale bar = 20 μm. **(P)** Quantification of the EdU^+^ cell ratio in O (n = 10). **(Q)** Representative images of migrated RAW 264.7 cells transfected with si-Ctrl or si-Dab1. Scale bar = 20 μm. **(R)** Quantification of migrated RAW 264.7 cells in Q (n = 10). **(S)** Representative EdU staining of RAW 264.7 cells transfected with si-Ctrl or si-Dab1. Scale bar = 20 μm. **(T)** Quantification of the EdU^+^ cell ratio in S (n = 10). **(U)** Representative immunofluorescence images showing Dab1, F4/80, and DAPI in the indicated groups. Scale bar = 200 μm. **(V)** Line-scan intensity profiles showing the spatial overlap of Dab1 and F4/80 signals along the indicated regions in U. Data are presented as mean ± SEM. Statistical analysis: one-way ANOVA with Tukey’s post hoc test as indicated. Statistical significance was determined as indicated (^**^*P* < 0.01, ^***^*P* < 0.001, ^****^*P* < 0.0001; ns, not significant).

**Figure S4. NHE1 functions as a downstream effector of Dab1 in macrophage HIF-1α signaling.** **(A)** GO enrichment analysis of differentially expressed proteins between Dab1-Ctrl and Dab1-cKO groups. Enriched terms are presented as –log_10_(*P* value). **(B)** Quantification of relative expression of NHE1, p-Dab1, and Dab1 protein levels in Figure 5F (n = 3). **(C-E)** Quantification of relative expression of HIF-1α, NHE1, p-Dab1, and Dab1 in Figure 5G-I (n = 3). **(F)** Quantification of NHE1 protein levels normalized to Na^+^/K^+^-ATPase in Figure 5L (n = 3). Data are presented as mean ± SEM. Statistical analysis: one-way ANOVA with Tukey’s post hoc test, as indicated. Statistical significance was determined as indicated (^*^*P* < 0.05, ^**^*P* < 0.01, ^***^*P* < 0.001, ^****^*P* < 0.0001; ns, not significant).

**Figure S5. Dab1 promotes vascular regeneration via the NHE1/HIF-1α pathway. (A)** Quantification of relative expression of CD31, HIF-1α, NHE1, p-Dab1, Dab1, and CD68 in Figure 6D (n = 3). **(B)** Quantification of relative expression of CD31, HIF-1α, NHE1, p-Dab1, Dab1, and CD68 in Figure 6H (n = 3). Data are presented as mean ± SEM. Statistical analysis: one-way ANOVA with Tukey’s post hoc test, as indicated. Statistical significance was determined as indicated (^*^*P* < 0.05, ^**^*P* < 0.01, ^***^*P* < 0.001, ^****^*P* < 0.0001; ns, not significant).

**Figure S6. Dab1 and its downstream targets are activated in human PNI tissues.** **(A)** Quantification of relative expression of HIF-1α, NHE1, p-Dab1, Dab1, and CD68 in Figure 7B (n = 3). **(B)** Quantification of relative expression of HIF-1α, p-Dab1, and Dab1 in Figure 7F (n = 3). Data are presented as mean ± SEM. Statistical analysis: one-way ANOVA with Tukey’s post hoc test as indicated. Statistical significance was determined as indicated (^*^*P* < 0.05, ^**^*P* < 0.01, ^***^*P* < 0.001, ^****^*P* < 0.0001; ns, not significant).

**Figure S7.** **Early valproic acid treatment promotes peripheral nerve regeneration and functional recovery.** **(A, B)** Quantification of axon diameter and myelin thickness in Figure 8H. **(C)** Scatter plot of G-ratio *versus* axon diameter in the indicated groups in Figure 8H, only the Sham and vehicle groups are shown here for clarity. Data are presented as mean ± SEM. Statistical analysis: one-way ANOVA with Tukey’s post hoc test as indicated. Statistical significance was determined as indicated (^*^*P* < 0.05, ^***^*P* < 0.001, ^****^*P* < 0.0001).

**Table S1-S6**

**Table S1 Patient information**

| Category | Patient ID | Sex (M/F) | Age（years） | Sample detail |
| --- | --- | --- | --- | --- |
| Injured nerves（PNI） | #1 | M | 36 | Right tibial nerve |
|  | #2 | F | 21 | Left median nerve |
|  | #3 | M | 35 | Left ulnar nerve |
| Non-injured nerves  (Control) | #4 | F | 16 | Right digital nerve |
|  | #5 | F | 12 | Right digital nerve |
|  | #6 | M | 8 | Left digital nerve |

**Table S2. Antibodies**

| **Antibodies** | **Source** | **Identifier** |
| --- | --- | --- |
| Rabbit Anti-S100β | Abcam | Cat#ab52642 |
| Mouse Anti-S100β | Sigma | Cat#S2532 |
| Rabbit Anti-NF-L | Cell Signaling Technology | Cat#2837 |
| Mouse Anti-NF-200 | Sigma | Cat#N5389 |
| Chicken Anti-F4/80 | Abcam | Cat#ab320060 |
| Rat Anti-CD31 | R&D | Cat#AF3628 |
| Rabbit Anti-CD31 | Cell Signaling Technology | Cat# 77699 |
| Mouse Anti-β-Actin | Transgen | Cat#HC201 |
| Mouse Anti-GAPDH | Transgen | Cat#HC301 |
| Rabbit Anti-Dab1 | Sigma | Cat# AB5840 |
| Chicken Anti-NeuN | Aveslab | Cat# C-2127-100 |
| Rabbit Anti-ATF3 | Abcam | Cat#ab207434 |
| Rabbit Anti- Phospho -Dab1 | Affinity | Cat#AF3029 |
| Mouse Anti-HIF-1α | Invitrogen | Cat# MA1516 |
| Rabbit Anti-NHE1 | Abcam | Cat# ab67314 |
| Rabbit Anti-Na^+^/K^+^-ATPase | ABclonal | Cat#A24883 |
| Rabbit Anti- CD68 | Abcam | Cat#ab283654 |
| Mouse Anti- CD68 | Invitrogen | Cat#MA5-13324 |
| Alexa Fluor 488 Donkey Anti-Rat | Invitrogen | Cat#A21208 |
| Alexa Fluor 647 Donkey Anti-Chicken | Invitrogen | Cat# A78952 |
| Alexa Fluor 488 Donkey Anti-Mouse | Invitrogen | Cat#A32766 |
| Alexa Fluor 488 Donkey Anti-Rabbit | Invitrogen | Cat#A11008 |
| Alexa Fluor 546 Donkey Anti-Mouse | Invitrogen | Cat#A10040 |
| Alexa Fluor 546 Donkey Anti-Rabbit | Invitrogen | Cat#A11035 |
| Alexa Fluor 647 Goat Anti-Mouse | Invitrogen | Cat#A31571 |
| HRP-Donkey-anti-rabbit | Beyotime | Cat#A0208 |
| HRP-Donkey-anti-mouse | Beyotime | Cat#A0216 |
| HRP-Donkey-anti-Rat | Beyotime | Cat# A0192 |

**Table S3. qPCR primers**

| **qPCR primer** | **Sequence** |
| --- | --- |
| β-Actin-F | CATTGCTGACAGGATGCAGAAGG |
| β-Actin-R | TGCTGGAAGGTGGACAGTGAGG |
| Dab1-F | GTGCTGTGACCCAATTAGAAC |
| Dab1-R | GACGGGAGAAAGGCATCACC |
| VEGF-A-F | CTGCTGTAACGATGAAGCCCTG |
| VEGF-A-R | GCTGTAGGAAGCTCATCTCTCC |

**Table S4. siRNA Sequences**

| Dab1 | Sense | 5’-GGGAGACAAGUUAUGUCAAGA-3 |
| --- | --- | --- |
|  | Antisense | 5’-UUGACAUAACUUGUCUCCCCG-3’ |
| NHE1 | Sense | 5'-GAGUGCAGAUUUCUAGCCCAUUUCA-3' |
|  | Antisense | 5'-UGAAAUGGGCUAGAAAUCUGCACUC-3' |
| Negative | Sense | 5’-UUCUCCGAACGUGUCACGUTT-3 |
|  | Antisense | 5’-ACGUGACACGUUCGGAGAATT-3’ |

**Table S5. Chemicals, peptides, recombinant proteins, and reagents**

| calcium peroxide (CaO₂) | Qian Yang BioTech | Cat# QY-CA004-F |
| --- | --- | --- |
| Calcium Hydroxide(Ca(OH)_2_) | Sigma | Cat# 450146 |
| DMEM/F12 | Bioscience | Cat#3130-0500 |
| DMEM | Sigma | Cat#D6429 |
| ECM | Sciencecell | Cat #1001 |
| Pluronic F127 | Sigma | Cat# P2443 |
| Corn Oil | MedChemExpres | Cat#HY-Y1888 |
| Opti-MEM | Gibco | Cat#31985-070 |
| Reelin | MedChemExpress | Cat# HY-P72276 |
| 30KDa centrifugal filter unit | Millipore | Cat# UFC9030 |
| Tamoxifen | MedChemExpress | Cat#HY-13757A |
| α-Bungarotoxin | BTPROBES | Cat#90-1022 |
| BCA | Beyotime | Cat# P0010 |
| DAPI | Genview | Cat#GD3410 |
| Hochest 3342 | Solarbio | Cat#23491 |
| Fetal Bovine Serum | Sigma | Cat#F8318 |
| 1XPBS | Bioss | Cat#C01-01001 |
| N2-supplyment | Gibco | Cat#17502-048 |
| 1XTBST | Servicebio | Cat#G2150 |
| Matrigel | Corning | Cat#354263 |
| Protease inhibitor mixture | Sangon Biotech | Cat# C50008 |
| BI-9627 | MedChemExpress | Cat# HY-18071 |
| Rosmarinic acid | MedChemExpress | Cat# HY-N0529 |
| Lipofectamine 3000 | Invitrogen | Cat#L3000150 |
| Bovine serum Albumin | Genview | Cat#FA016 |
| Donkey serum | Servicebio | Cat#G1217 |
| Goat serum | Servicebio | Cat#G1208 |
| DMSO | Sigma | Cat#D2650 |
| GlutMAX | Gibco | Cat#35050061 |
| Penicillin-Streptomycin Solution | Beyotime | Cat#C0222 |
| Wheat Germ Agglutinin (WGA) | Maokangbio | Cat#MP6325 |
| Tween-20 | Ding Guo | Cat#DH358-3 |
| Tween-80 | Ding Guo | Cat#DH360-3 |
| PEG-300 | Shyuanye | Cat#25322-68-3 |
| TritonX-100 | Ding Guo | Cat#DH351-4 |
| Western Blot Antibody Stripping Solution | Epizyme | Cat#PS107 |

**Table S6. Critical commercial assays**

| Hypoxyprobe™-1 Kit | Hypoxyprobe | Cat# HP1-100Kit |
| --- | --- | --- |
| Apollo EdU Imaging Kit | Ribo Biotechnology | Cat# C10310-1 |
| TB Green™ Premix Ex Taq™ | TaKaRa | Cat#RR420A |
| TaKaRa MiniBEST Universal RNA Extraction kit | TaKaRa | Cat#9767 |
| Transwell | Corning | Cat#REF3422 |
| ELISA VEGF-A Assay Kit | MEIMIAN | Cat#MM-44452M1 |
